# miR-322/-503 rescues myoblast defects in myotonic dystrophy type 1 cell model by targeting CUG repeats

**DOI:** 10.1038/s41419-020-03112-6

**Published:** 2020-10-22

**Authors:** Xiaopeng Shen, Feng Xu, Meng Li, Shen Wu, Jingyi Zhang, Ao Wang, Lei Xu, Yu Liu, Guoping Zhu

**Affiliations:** 1 School of Life Sciences, Anhui Normal University, Wuhu, China; 2 The Key Laboratory of Biomedicine in Gene Diseases and Health of Anhui Higher Education Institutes, Anhui Normal University, Wuhu, China; 3 Anhui Province Key Laboratory of Active Biological Macromolecules, Wannan Medical College, Wuhu, China; 4 Department of Biology and Biochemistry, University of Houston, Houston, TX USA

**Keywords:** Cell biology, Diseases

## Abstract

Myotonic dystrophy type 1 (DM1) is the most common type of adult muscular dystrophy caused by the expanded triple-nucleotides (CUG) repeats. Myoblast in DM1 displayed many defects, including defective myoblast differentiation, ribonuclear foci, and aberrant alternative splicing. Despite many were revealed to function in DM1, microRNAs that regulated DM1 via directly targeting the expanded CUG repeats were rarely reported. Here we discovered that miR-322/-503 rescued myoblast defects in DM1 cell model by targeting the expanded CUG repeats. First, we studied the function of miR-322/-503 in normal C2C12 myoblast cells. Downregulation of miR-322/-503 significantly hindered the myoblast differentiation, while miR-322/-503 overexpression promoted the process. Next, we examined the role of miR-322/-503 in the DM1 C2C12 cell model. miR-322/-503 was downregulated in the differentiation of DM1 C2C12 cells. When we introduced ectopic miR-322/-503 expression into DM1 C2C12 cells, myoblast defects were almost fully rescued, marked by significant improvements of myoblast differentiation and repressions of ribonuclear foci formation and aberrant alternative splicing. Then we investigated the downstream mechanism of miR-322/-503 in DM1. Agreeing with our previous work, Celf1 was proven to be miR-322/-503′s target. Celf1 knockdown partially reproduced miR-322/-503′s function in rescuing DM1 C2C12 differentiation but was unable to repress ribonuclear foci, suggesting other targets of miR-322/-503 existed in the DM1 C2C12 cells. As the seed regions of miR-322 and miR-503 were complementary to the CUG repeats, we hypothesized that the CUG repeats were the target of miR-322/-503. Through expression tests, reporter assays, and colocalization staining, miR-322/-503 was proved to directly and specifically target the expanded CUG repeats in the DM1 cell model rather than the shorter ones in normal cells. Those results implied a potential therapeutic function of miR-322/-503 on DM1, which needed further investigations in the future.

## Introduction

Myotonic dystrophy type 1 (DM1) is a dominant autosomal inherited neuromuscular disease. DM1 is caused by the expanded triple-nucleotides (CTG) repeats in the 3′UTR of the *DMPK* gene. The CTG repeats are transcribed into the expanded CUG repeats, called the toxic RNA^1^. The toxic RNA forms a hairpin-like secondary structure, resulting in MBNL1 sequestration and Celf1 upregulation. MBNL1 sequestration leads to ribonuclear foci formation and lowered functional MBNL1 levels^2^. Unlike MBNL1, the Celf1 level was enhanced by the PKC mediated hyperphosphorylation^3^ and the decrease of miR-23a/b^4^. The aberrant levels of MBNL1 and Celf1 in DM1 caused aberrant splicing patterns of many genes, such as *CLCN1*, *IR*, *PKM*, and *TNNT2*, resulting in disease phenotypes^5–10^. Loss of MBNL1 or gain of Celf1 in mouse partially recaptured aberrant alternative splicing, muscle wasting, and defective heart function in DM1^11–14^. Celf1 was reported to promote myogenic factor (Mef2A and p21) expressions^15,16^ but significantly hindered myogenesis process^17^.

Currently, many targeted therapy strategies against DM1 were proposed. Ectopic MBNL1 expression by AAV infection or repression of Celf1 hyperphosphorylation by PKC inhibition were promising ways^18,19^. Another more radical strategy was directly targeting the expanded CUG repeats, which included degrading the CUG repeats via RNA interference and small chemical molecules^20,21^, or blocking the binding of RNA binding factors to the CUG repeats using small molecules and peptides^22^. Moreover, removal of the CTG repeats in the genome by CRISPR/Cas9 reverted ribonuclear foci formation and aberrant splicing pattern in DM1^23,24^. Despite those potential therapy strategies, microRNAs (miRNAs) involved therapy strategies were rarely reported^25–27^.

miRNAs are a group of 18~22 nucleotides noncoding RNAs, which are incorporated with RNA induced silencing complex to downregulate target mRNAs. The recognition of miRNAs to their target mRNAs is mainly determined by the fully complementary binding of miRNAs’ seed regions to the targets. Previously many miRNAs were discovered to participate in DM1 pathology and therapy, such as miR-1^28,29^, miR-206^27,30^, miR-148a^30^, and miR-15b/16^30^. Among those, miR-206 and miR-148a were reported to directly target non-CUG repeat region of DMPK 3′UTR, while miR-15b/16 were shown to target the CUG repeat region based on molecular and biochemistry data, leaving biological functions in DM1 undetermined^30^. Together, previously reported miRNAs regulated DM1 mainly through manipulating MBNL1 and Celf1. The miRNAs that rescue DM1 defects by directly targeting the expanded CUG repeats were rarely reported to our best knowledge.

miR-322/-503, a miRNA cluster on X chromosomes of mouse and human, is consisted of miR-322 (miR-424 in human) and miR-503. The expressions of both miRNAs are under the control of the same *cis*-elements. miR-322/-503 was reported to function mainly in cancer^31–33^, angiogenesis^34,35^, cardiovascular diseases^36–38^, and development fields^39–41^. Our previous study demonstrated that miR-322/-503 was enriched in early cardiac progenitors and promoted cardiac differentiation by repressing Celf1^39^. As to myoblast differentiation, miR-322/-503’s role was under debate: one reference claimed that miR-322/-503 promoted myoblast differentiation by inhibiting cell cycle via targeting Cdc25A^40^, while another one argued that miR-322 inhibited myoblast differentiation by targeting SETD3^41^. Moreover, although miR-322/-503 was proven to target Celf1 during cardiac differentiation, miR-322/-503’s function in DM1 was still illusive.

In this study, we discovered that miR-322/-503 could rescue myoblast defects by directly targeting the expanded CUG repeats in the DM1 cell model. Through gain-and loss-of-function analysis, we found that miR-322/-503 was essential in normal myoblast differentiation. Ectopic miR-322/-503 expression rescued defective myoblast differentiation, ribonuclear foci formation, and aberrant alternative splicing by directly targeting not only Celf1 but the expanded CUG repeats in the DM1 C2C12 cell model.

## Materials and methods

### Cell culture and flow cytometry

C2C12 and HEK293T cells were purchased from Stem Cell Bank, Chinese Academy of Sciences, which were authenticated by STR profiling and free of mycoplasma contamination.

C2C12 cells were cultured in the growth medium (namely GM), which was the Dulbecco’s Modified Eagle’s Medium-high glucose (HyClone, UT, USA) supplemented with 20% Fetal Bovine Serum (Clark Bioscience, VA, USA), 50 U/mL penicillin (Gibco, NY, USA), and 50 μg/mL streptomycin (Gibco). C2C12 cells differentiation were induced by switching medium from GM to the differentiation medium (DM), which was the Dulbecco’s Modified Eagle’s Medium-high glucose (HyClone) supplemented with 2% Horse Serum (Gibco), 50 U/mL penicillin, 50 μg/mL streptomycin (Gibco) and 1 μM insulin (Beyotime, Shanghai, China). To perform C2C12 differentiation, C2C12 cells were seeded onto 6-well plates and initially cultured in GM. When confluent, C2C12 cells were switched to culturing in DM for another 6 days, which were named as Days 1–6. Samples were collected at Day 0 (in GM), 1, 2, 4, 6, except where otherwise stated.

HEK293T cells were cultured in the Dulbecco’s Modified Eagle’s Medium-high glucose (HyClone) supplemented with 10% Fetal Bovine Serum (Clark Bioscience), 50 U/mL penicillin (Gibco), and 50 μg/mL streptomycin (Gibco).

When doing flow cytometry to detect GFP expression, cells were trypsin digested into single cells and examined by FACSCanto II (BD Biosciences, CA, USA). All data were analyzed with FlowJo v10 software.

### Plasmids and cell line constructions

pLL4.0 vector was developed by replacing the GFP coding region in pLL3.8 vector with puromycin resistance coding gene. pCW57-MCS1-2A-MCS2 plasmid as a Doxycycline inducible vector was purchased from Addgene. The miR-322/-503 coding fragment was polymerase chain reaction (PCR) amplified from mouse genome with the primers: forward, 5′-CTCGAGGAATTCGCTGGCAAGAGTGATCCAGATGTT-3′; reverse, 5′- CTCGAGGAATTCTTTACCTGAGCAGCAAGTGAGGC-3′. The miR-322/-503 fragment was digested with EcoRI and ligated into pLL4.0 and pCW57-MCS1-2A-MCS2 vectors at the EcoRI site to produce pLL4.0-miR-322/-503 and pCW57-miR-322/-503 plasmids. The plasmids were sequenced to confirm their validity. pcDNA-GFP-(CUG)_5_ and pcDNA-GFP-(CUG)_200_ (short for GFP-CUG5 and GFP-CUG200), as reported previously^42^, contained GFP expression cassettes that were in conjunction with 5 and 200 copies of CTG repeats in their 3′UTR regions. miRZip-424 and miRZip-503 (System Biosciences, CA, USA) were purchased and used as the inhibitors of miR-322 (miR-424 ortholog in mouse) and miR-503. The control plasmid for miRZip was obtained from the same source. Celf1 shRNA (TRCN0000098511) and its corresponding scramble control plasmids were commercially available (GE Healthcare, IL, USA).

Plasmid transfections were performed using lipofectamine 2000 (Invitrogen, CA, USA). Before plasmid transfections, both C2C12 and HKE293T cells were seeded one day ahead to make their confluence to be 80–90% for HKE293T cells and 40–50% for C2C12 cells at transfection. The ratio of plasmids to lipofectamine 2000 was 1:2 (μg:μL). The cell culture medium was changed 24 h after transfection. G418 or puromycin selections were performed 48 h after transfection if stable cell lines were needed.

C2C12/mirKD-322, C2C12/mirKD-503, and C2C12/mirKD-Ctrl cell lines were produced by stably transfecting C2C12 myoblast cells with mirZIP-424, miR-503, and the control plasmid for miRZip, respectively. C2C12/control and C2C12/miR-322/-503 cell lines were produced by stably transfecting C2C12 myoblast cells with pLL4.0 and pLL4.0-miR-322/-503 plasmids, respectively. normal (C2C12-CUG5) and DM1 (C2C12-CUG200) myoblast cell models were produced by stably transfecting C2C12 cells with GFP-CUG5 and GFP-CUG200 plasmids, respectively. C2C12-CUG5/control and C2C12-CUG5/miR-322/-503 cell lines were produced by stably transfecting C2C12-CUG5 cells with pLL4.0 and pLL4.0-miR-322/-503 plasmids, respectively. C2C12-CUG200/control and C2C12-CUG200/miR-322/-503 cell lines were produced by stably transfecting C2C12-CUG200 cells with pLL4.0 and pLL4.0-miR-322/-503 plasmids, respectively. C2C12-CUG200/scramble and C2C12-CUG200/shCelf1 cell lines were produced by stably transfecting C2C12-CUG200 cells with scramble control and Celf1 shRNA plasmids, respectively. C2C12-CUG5/pCW57-miR-322/-503 and C2C12-CUG200/pCW57-miR-322/-503 cell lines were produced by stably transfecting C2C12-CUG5 and C2C12-CUG200 cells with pCW57-miR-322/-503 plasmid.

### Luciferase assays

Luciferase assay vectors to test if miR-322/-503 targeted Celf1’s 3′UTR were constructed as previously reported^39^. Celf1-3′UTR was constructed by ligating the nucleotides 1661~2261 region of Celf1’ 3′UTR, which contained the predicted miR-322/-503 mutual binding sites, into pmirGLO vector (Promega, WI, USA) using In-Fusion HD Cloning kit (Clontech, CA, USA). Celf1-3′UTR-mut was constructed by replacing the predicted miR-322/-503 seed regions’ binding sequence-“GACTGCT” with “CTGACGA”. The luciferase assays were performed utilizing the Dual-Luciferase Reporter Assay System according to the manufacturer′s protocol. (Promega)

### Total RNA extraction and real-time quantitative PCR (RT-qPCR)

Total RNA samples were extracted using Total RNA Isolation Reagent (Biosharp, Hefei, China). For protein-coding gene expression quantification, reverse transcriptions were performed using FastKing RT Kit (Tiangen, Beijing, China) and Quantitative PCRs were performed using Powerup SYBR Master Mix (Applied Biosystems, CA, USA). GAPDH served as a normalized control. As to microRNA, cDNA samples were produced sequentially by adding poly(A) tail with *E.coli* Poly(A) Polymerase (New England Biolabs, MA, USA) and doing reverse transcription with SuperScript™ III Reverse Transcriptase (Invitrogen) and a universal reverse transcription primer (5′-CAGGTCCAGTTTTTTTTTTTTTTTVN-3′; “V” stands for A, C, or G, and “N” stands for A, T, C, or G.) as described previously^43–45^. Quantitative PCRs for microRNA were performed utilizing Powerup SYBR Master Mix (Applied Biosystems). microRNAs′ expression was relative to U6. All quantitative PCR primer sequences are provided in the supplement (Table S1).

### Western Blot

Cells were lysed in the Cell Lysis Buffer (Byotime) with protease inhibitors (Roche, Basel, Switzerland). Protein concentrations were measured using the BCA protein assay kit (Biosharp) and adjusted to the same in each experiment set. Proteins were subjected to the electrophoresis on SDS-PAGE gels, which were consisted of 5% stacking gel and 10% separation gel (15% separation gel for LC3B western blots). The proteins then were transferred onto PVDF membranes. The membranes were blocked and incubated with primary antibodies overnight. On the next day, the membranes were incubated with secondary antibodies and reacted with chemiluminescent substrates (Biosharp) to produce signals. The signals were captured by scanning with Tanon 5200 Imaging system (Tanon, Shanghai, China). The antibodies and their dilutions used were as follows: CELF1 mAb (1:1000, Santa Cruz, TX, USA, Catalog No. sc-20003), GAPDH mAb (1:2000, ZSGB-BIO, Beijing, China, Catalog No.17AF0406), GFP mAb (1:3000, Sino Biological, Beijing, China, Catalog No. 13105-R208), LC3B mAb (1:1000, Abcam, Cambridge, UK, Catalog No. A7198), goat anti-mouse HRP-conjugated IgG (1:2000, Biosharp, Catalog No. BL001A), and donkey anti-rabbit HRP-conjugated IgG (1:2000, Invitrogen, Catalog No. 31458).

### Immunostaining

Cells on slides or tissue culture plates were fixed with 4% paraformaldehyde (PFA) for MF-20 immunostaining, whereas cells were fixed with chilled methanol: acetone (1:1) mixed solution in 4° when doing MBNL1 immunostaining to detect ribonuclear foci. Following fixation, cells were blocked with the blocking solution (10% normal goat serum, 0.1% Triton X-100 in phosphate-buffered saline (PBS)). The cells were then incubated overnight in primary antibodies. On the next day, the cells were incubated in the fluorescence conjugated-secondary antibodies for 90 min at room temperature and stained with DAPI for 5 min. MF-20 immunostaining images were taken with an Olympus fluorescence microscope. MBNL1 immunostaining images were taken with a Zeiss ApoTome.2 fluorescence microscope. The antibodies and their dilutions used were as following: MF-20 mAb (1:10, DSHB, IA, USA, Catalog No. AB_2147781), MBNL1 mAb (1:100, Novus, CO, USA, Catalog No. NB110-37256), goat anti-mouse Alex Fluor 488-conjugated IgG (1:500, Invitrogen, Catalog No. A11001), and goat anti-mouse Alex Fluor Plus 555-conjugated IgG (1:500, Invitrogen, Catalog No. A32727). Immunostaining images were analyzed by ImageJ2X software. The fusion index was the ratio of nuclei number in the cells with at least two nuclei versus total nuclei number. Myotube area was calculated as the ratio of the MF20 fluorescence positive area versus the whole image area in the immunostaining images. Fluorescence intensity was calculated as the ratio of total fluorescence intensity within cells vs. the total cell area.

### RNA fluorescence in situ hybridization (RNA FISH)

RNA FISH was performed according to the protocol described previously^30^. Cells were fixed with 4% PFA at 4° for 20 min. Following fixation, the cells were washed three times with PBS and permeabilized with 0.5% Triton X-100 in PBS supplemented with 2 mM ribonucleoside vanadyl complex (RVC) for 7 min. Next, the cells were incubated in 30% formamide and 2× SSC for 10 min. As to hybridization, the cells were incubated in the hybridization buffer (30% formamide, 2× SSC, 0.02% bovine serum albumin, 66 µg/ml yeast tRNA, 10% dextran sulfate, 2 mM RVC, and 2 ng/µl probes) for 24 h. Following hybridization, the cells were washed with 30% formamide and 2× SSC at 45° for 30 min and then 1× SSC at 37° for another 30 min. The cells were mounted in Antifade Mounting Medium with DAPI (Beyotime) and subjected to observation and image capture using a Zeiss ApoTome.2 fluorescence microscope. Probes used in the study were as following: CAG probe for the expanded CUG repeats detection, 5′-CAGCAGCAGCAGCAGCAGCAG-3′ with 5′-FAM label and 2′-O-methyl modification at the first two nucleotides; miR-322 probe for miR-322 detection, 5′-TCCAAAACATGAATTGCTGCT-3′ with 5′-Cy3 label; miR-503 probe for miR-503 detection, 5′-AGTACTGTTCCCGCTGCTA-3′ with 5′-Cy3 label. RNA FISH images were analyzed by the Colocalization plugin in ImageJ2X software. The colocalization ratio of miR-322/-503 and the expanded CUG repeats was calculated as the ratio of miRNAs and the CUG repeats dual-positive area versus the area that was at least one probe positively stained.

### β-galactosidase staining

Female heterozygous miR-322/-503 LacZ knock-in mice were generated by initial mating male Mirc24^tm1Mtm^/Mmjax (MMRRC Stock No: 36306-JAX) mice with female Tg(Sox2-Cre) mice and further mating with other wild-type mice. E10.5 embryos, which were LacZ genotyping positive, were collected and fixed for 30 min in 4% PFA. The embryos were then washed three times with wash buffer, which was PBS supplemented with 0.02% NP-40 and 0.01% sodium deoxycholate. Afterwards, the embryos were stained with staining solution, which was PBS supplemented with 5 mM K_3_Fe(CN)_6_, 5 mM K_4_Fe(CN)_6_, 0.02% NP-40, 0.01% sodium deoxycholate, 2 mM MgCl_2_, 5 mM EGTA, and 1 mg/ml X-gal. The embryos were transferred to wash buffer after specific staining appeared and ready for image capture. All mice related experimental procedures were approved by the Institutional Animal Care and Use Committee (IACUC).

### RT-PCR for alternative splicing test

Total RNA samples were extracted using Total RNA Isolation Reagent (Biosharp). The RNA samples were reverse transcribed by RevertAid Reverse Transcriptase (Thermo Fisher, MA, USA) to produce cDNA. cDNA samples were then amplified by Phusion Hot Start II High-Fidelity PCR Master Mix (Thermo Fisher) with designed primers, the sequences of which were provided in the supplement (Table S2). The PCR products were subjected to the electrophoresis on 3% agarose gel and scanned with the Tanon gel image system 1600. The densities of bands on gel images were measured by Tanon gel image system integrated software.

### Statistical analysis

Three biological replicates and three technical replicates were performed for all assays except where otherwise stated. Significance was determined by the two-sided *t* test and *p* < 0.05 was considered to be statistically significant. All data were presented as mean ± SD.

## Results

### miR-322/-503 was required in myoblast differentiation

**Fig. 1:**
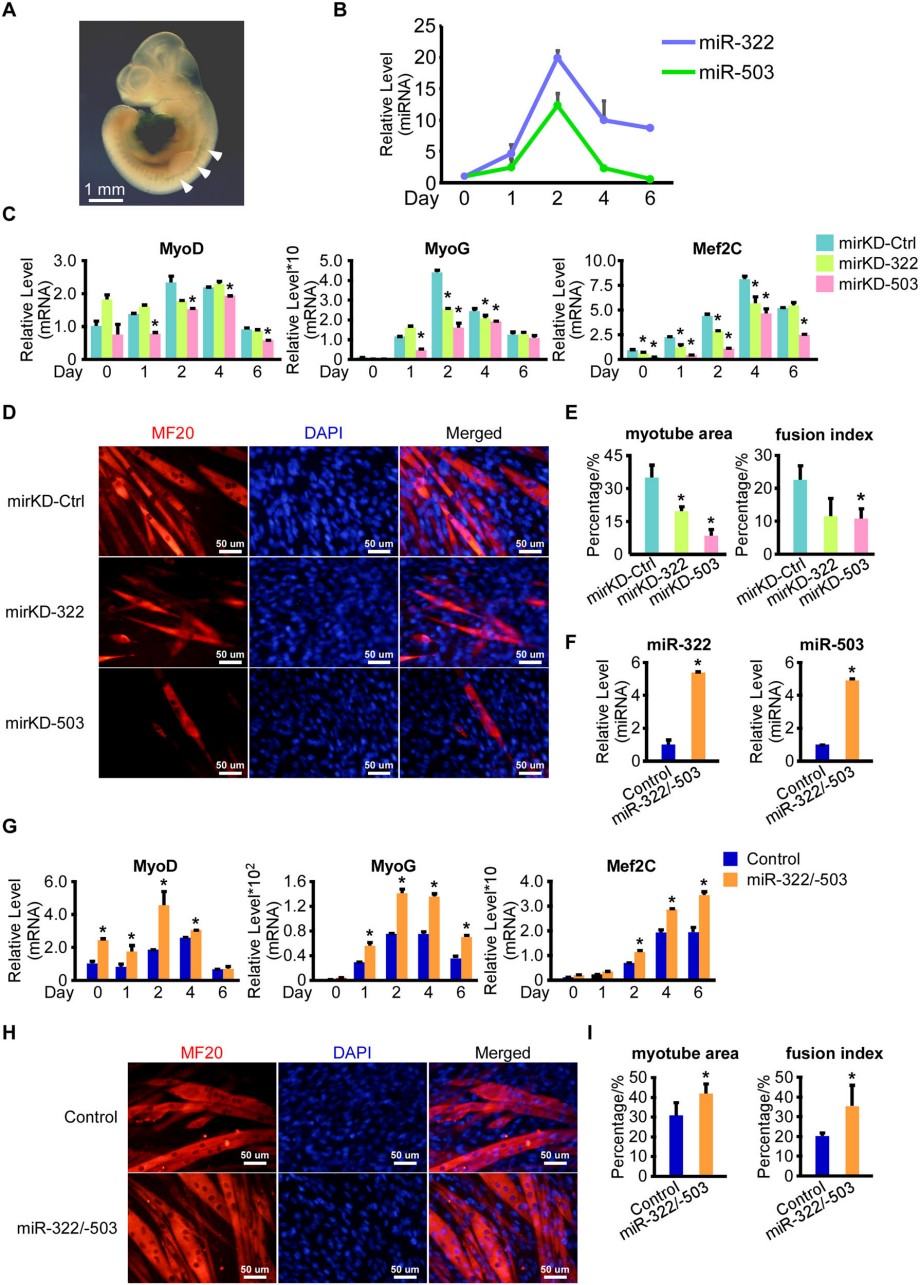
miR-322/-503 was required in myoblast differentiation. **A** miR-322/-503 was specifically expressed at the heart region and somites in E10.5 mouse embryos, which was determined by beta-galactosidase staining. White arrows pointed to the somites staining. **B** Expression patterns of miR-322 and miR-503 during normal myoblast differentiation. The expression levels of miR-322 and miR-503 were normalized to their day 0 values. **C** Inhibitors of miR-322 and miR-503 significantly repressed the expressions of myogenic markers (MyoD, MyoG, and Mef2C). All expression levels were normalized to the control group at day 0. **D** Inhibitors of miR-322 and miR-503 impaired myotube formation. Myotube formation was displayed by immunostaining of MF20 on day 6. **E** Inhibitors of miR-322 and miR-503 decreased myotube areas (left) and fusion index (right) of myoblast differentiation. **F** Overexpression of miR-322/-503 was verified by RT-qPCR. The expression levels of miR-322 and miR-503 were normalized to the control group. **G** miR-322/-503 overexpression significantly upregulated the expressions of myogenic markers (MyoD, MyoG, and Mef2C). All expression levels were normalized to the control group at day 0. **H** miR-322/-503 overexpression promoted myotube formation. Myotube formation was displayed by immunostaining of MF20 on day 6. **I** miR-322/-503 overexpression increased myotube areas (left) and fusion index (right) of myoblast differentiation. mirKD-Ctrl, C2C12/mirKD-Ctrl cells; mirKD-322, C2C12/mirKD-322 cells; mirKD-503, C2C12/mirKD-503 cells; Control, C2C12/control cells; miR-322/-503, C2C12/miR-322/-503 cell lines; * statistically significant (*p* < 0.05).

miR-322/-503 was revealed to promote cardiac differentiation by targeting Celf1 in early cardiac progenitor cells^39^, but its function in myoblast differentiation was still under debate^40,41^. We performed β-galactosidase staining on miR-322/-503 lacZ knock-in mouse E10.5 embryos. miR-322/-503 was specifically expressed in the heart region and somites, suggesting that miR-322/-503 might be involved in not only the heart but the skeletal muscle developments (Fig. 1A). Then we performed myoblast differentiation of C2C12 cells and measured the expression patterns of miR-322 and miR-503. Both miR-322 and miR-503 displayed expression peaks at day 2, suggesting their elevation at day 2 might be needed to ensure the normal differentiation process (Fig. 1B).

To prove this, we performed a loss-of-function test of miR-322/-503 on normal myoblast differentiation. We performed differentiation on C2C12/mirKD-322, C2C12/mirKD-503, and C2C12/mirKD-Ctrl cell lines. Through RT-qPCR, MyoD was almost unaffected by mirKD-322 but significantly repressed by mirKD-503; MyoG and Mef2C expressions were dramatically repressed by both mirKD-322 and mirKD-503, whereas mirKD-503 displayed stronger repression effects than mirKD-322 (Fig. 1C). Compared to mirKD-Ctrl, mirKD-322, and mirKD-503 groups showed much fewer myotubes, marked by dramatically lowered myotube areas and fusion index with sarcomere myosin (MF20) immunostaining at day 6 (Fig. 1D, E). Compared to the mirKD-322 group, mirKD-503 formed even less myotube formation.

Next, we did a gain-of-function analysis of miR-322/-503 on normal myoblast differentiation. We constructed the control and miR-322/-503 overexpressing C2C12 cells (namely C2C12/control and C2C12/miR-322/-503 cells), which were subjected to differentiation. The overexpression of miR-322/-503 was verified by RT-qPCR (Fig. 1F). Myogenic markers (MyoD, MyoG, and Mef2C) were significantly upregulated with miR-322/-503 overexpression (Fig. 1G). Myotube formation was significantly increased by miR-322/-503 overexpression as well (Fig. 1H, I). Together, miR-322/-503 was essential for myoblast differentiation, which might be a causative mechanism for many muscular diseases in which miR-322/-503 was dysregulated.

### miR-322/-503 was dysregulated in the DM1 cell model

**Fig. 2:**
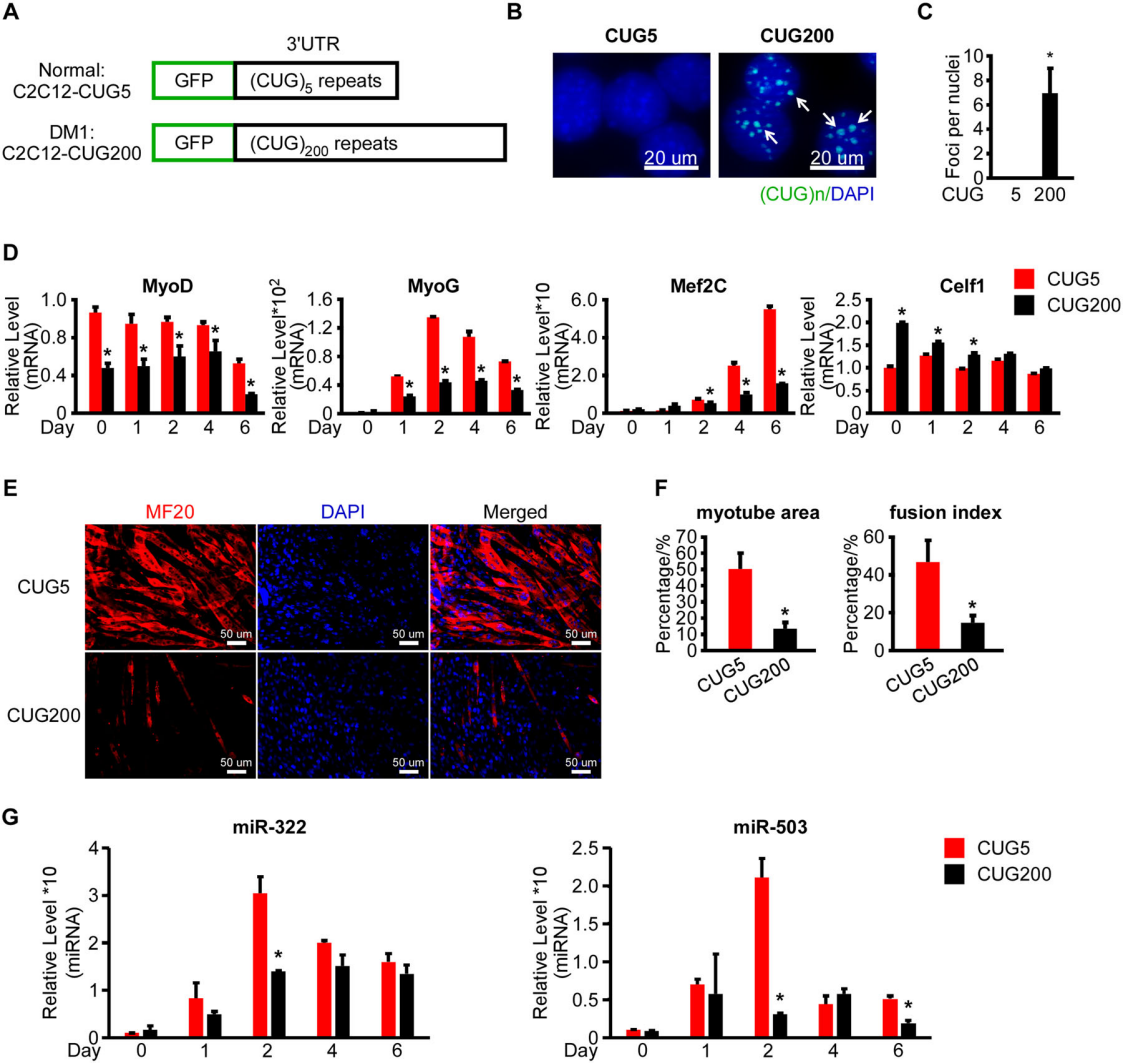
miR-322/-503 was dysregulated in myotonic dystrophy type 1. **A** Schematic diagram of GFP-CUG5 and GFP-CUG200. GFP-CUG5 was used to produce the normal C2C12 cell model (C2C12-CUG5) and GFP-CUG200 was used to produce the DM1 C2C12 model (C2C12-CUG200). **B**, **C** Ribonuclear foci were detected in C2C12-CUG200 cells with CAG probe RNA FISH, but not detected in C2C12-CUG5 cells. **D** Myogenic markers (MyoD, MyoG, and Mef2C) were significantly repressed but Celf1 was upregulated during myoblast differentiation of C2C12-CUG200 compared to C2C12-CUG5. All expression levels were normalized to the CUG5 group at day 0. **E** C2C12-CUG200 differentiation displayed impaired myotube formation compared to C2C12-CUG5. Myotube formation was displayed by immunostaining of MF20 on day 6. **F** C2C12-CUG200 differentiation showed decreased myotube areas (left) and fusion index (right) compared to C2C12-CUG5. **G** miR-322 and miR-503 were significantly downregulated at day 2 in C2C12-CUG200 differentiation. All expression levels were normalized to the CUG5 group at day 0. CUG5, C2C12-CUG5 cells; CUG200, C2C12-CUG200 cells; * statistically significant (*p* < 0.05).

In DM1, the myoblast differentiation capacity was still under debate. Most studies performed in cell models, mouse models, and patient-derived cells reported impaired myoblast differentiation in DM1^3,15,17,46^, though several groups noticed normal myoblast differentiation in the patient-derived cells of different origins^47,48^. Moreover, since miR-322/-503 was required by myoblast differentiation and it was reported to directly target Celf1 in cardiac differentiation^39^, we determined to study the myoblast differentiation pattern and the expression profiles of miR-322/-503 in DM1 C2C12 cell model. Firstly, we constructed normal (C2C12-CUG5) and DM1 (C2C12-CUG200) myoblast cell models by stably transfecting C2C12 cells with GFP-CUG5 and GFP-CUG200 plasmids, which transcribed 5-copy and 200-copy CUG repeats at the 3′UTR of GFP mRNA (Fig. 2A). Ribonuclear foci were detected in C2C12-CUG200 nuclei, but not in C2C12-CUG5 nuclei with RNA FISH and MBNL1 immunostaining, suggesting that the cell models were successfully constructed (Figs. 2B, C and S2A). We then performed differentiation on C2C12-CUG5 and C2C12-CUG200 cells. In agreement with previous reports^17,42,46^, myogenic markers (MyoD, MyoG, and Mef2C) were dramatically repressed in DM1, while the Celf1 level was significantly elevated (Fig. 2D). On day 6, the C2C12-CUG5 group displayed abundant myotubes, while the C2C12-CUG200 group had only sporadic ones (Fig. 2E). The myotube area was 50.2% ± 9.9% in C2C12-CUG5, but 13.3% ± 4.0% in C2C12-CUG200. Fusion index was 46.7% ± 11.5% in C2C12-CUG5, but 14.5% ± 3.9% in C2C12-CUG200 (Fig. 2F). By RT-qPCR, we found that miR-322/-503 was significantly repressed on day 2 in the C2C12-CUG200 group when miR-322/-503 displayed expression peaks during wild-type myoblast differentiation (Fig. 2G).

### miR-322/-503 rescued myoblast differentiation defects and repressed ribonuclear foci in the DM1 cell model

**Fig. 3:**
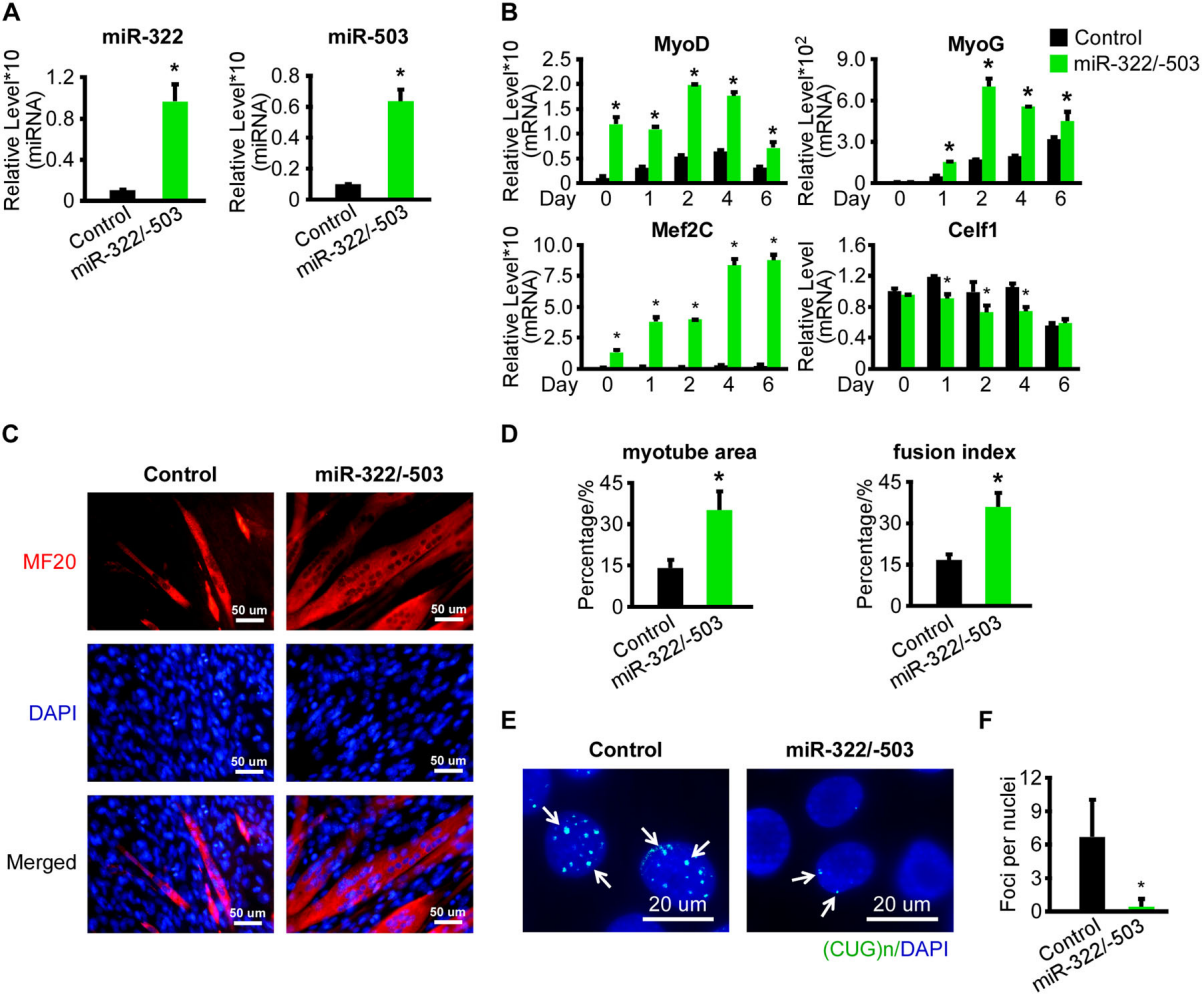
miR-322/-503 rescued myoblast differentiation defects and repressed ribonuclear foci in DM1. **A** Overexpression of miR-322/-503 in C2C12-CUG200 cells was verified by RT-qPCR. The expression levels of miR-322 and miR-503 were normalized to the control group. **B** miR-322/-503 overexpression significantly upregulated the expressions of myogenic markers (MyoD, MyoG, and Mef2C) and downregulated Celf1 during C2C12-CUG200 differentiation. All expression levels were normalized to the control group at day 0. **C** miR-322/-503 overexpression promoted myotube formation of C2C12-CUG200 differentiation. Myotube formation was displayed by immunostaining of MF20 on day 6. **D** miR-322/-503 overexpression increased myotube areas (left) and fusion index (right) of C2C12-CUG200 differentiation. **E**, **F** miR-322/-503 overexpression repressed ribonuclear foci formation in C212-CUG200 cell. The ribonuclear foci were detected with CAG probe RNA FISH. Control, C2C12-CUG200/control cells; miR-322/-503, C2C12-CUG200/miR-322/-503 cells; * statistically significant (*p* < 0.05).

To examine the miR-322/-503’s function in DM1, we constructed the control and miR-322/-503 overexpressing C2C12-CUG200 cells (namely C2C12-CUG200/control and C2C12-CUG200/miR-322/-503 cells), which were subjected to differentiation. The overexpression of miR-322/-503 in C2C12-CUG200/miR-322/-503 cells was confirmed by RT-qPCR (Fig. 3A). With miR-322/-503 overexpression, the expressions of myogenic markers (MyoD, MyoG, and Mef2C) were significantly improved, while Celf1 expression was inhibited (Fig. 3B). Through MF-20 immunostaining, miR-322/-503 overexpression in C2C12-CUG200 cells displayed identical myotube formation as C2C12-CUG5 group (Fig. 2E), while the C2C12-CUG200/control group still showed sporadic myotube formation (Fig. 3C), characterized by myotube area of 35.0%±6.9% with miR-322/-503 overexpression vs. 14% ± 3.1% in control and fusion index of 35.9% ± 5.2% with miR-322/-503 overexpression vs. 16.6% ± 2.1% in control (Fig. 3D). The values of myotube area and fusion index with miR-322/-503 overexpression were very close to those of the C2C12-CUG5 group (Fig. 2F). Moreover, we observed remarkable repression of ribonuclear foci formation in DM1 by miR-322/-503 overexpression via RNA FISH and immunostaining of MBNL1 (Figs. 3E, F and S2B). As ribonuclear foci formation was suppressed by miR-322/-503, we were wondering if the sequestered MBNL1 was released from the foci and whether the total MBNL1 level within cells was affected. Thus, when we took the immunofluorescence image of MBNL1, we also adjusted the fluorescence threshold to display not only aggregated MBNL1 in ribonuclear foci but diffused one. We noticed that the total MBNL1 fluorescence intensity was not altered with miR-322/-503 overexpression in C2C12-CUG200 cells, suggesting more diffused MBNL1 existed (Fig. S2D, E). Those results suggested that miR-322/-503 not only rescued myoblast differentiation defects but inhibited ribonuclear foci formation in DM1.

### miR-322/-503 targeted Celf1 leading to partial rescue of myoblast differentiation defects but not ribonuclear foci in DM1

**Fig. 4:**
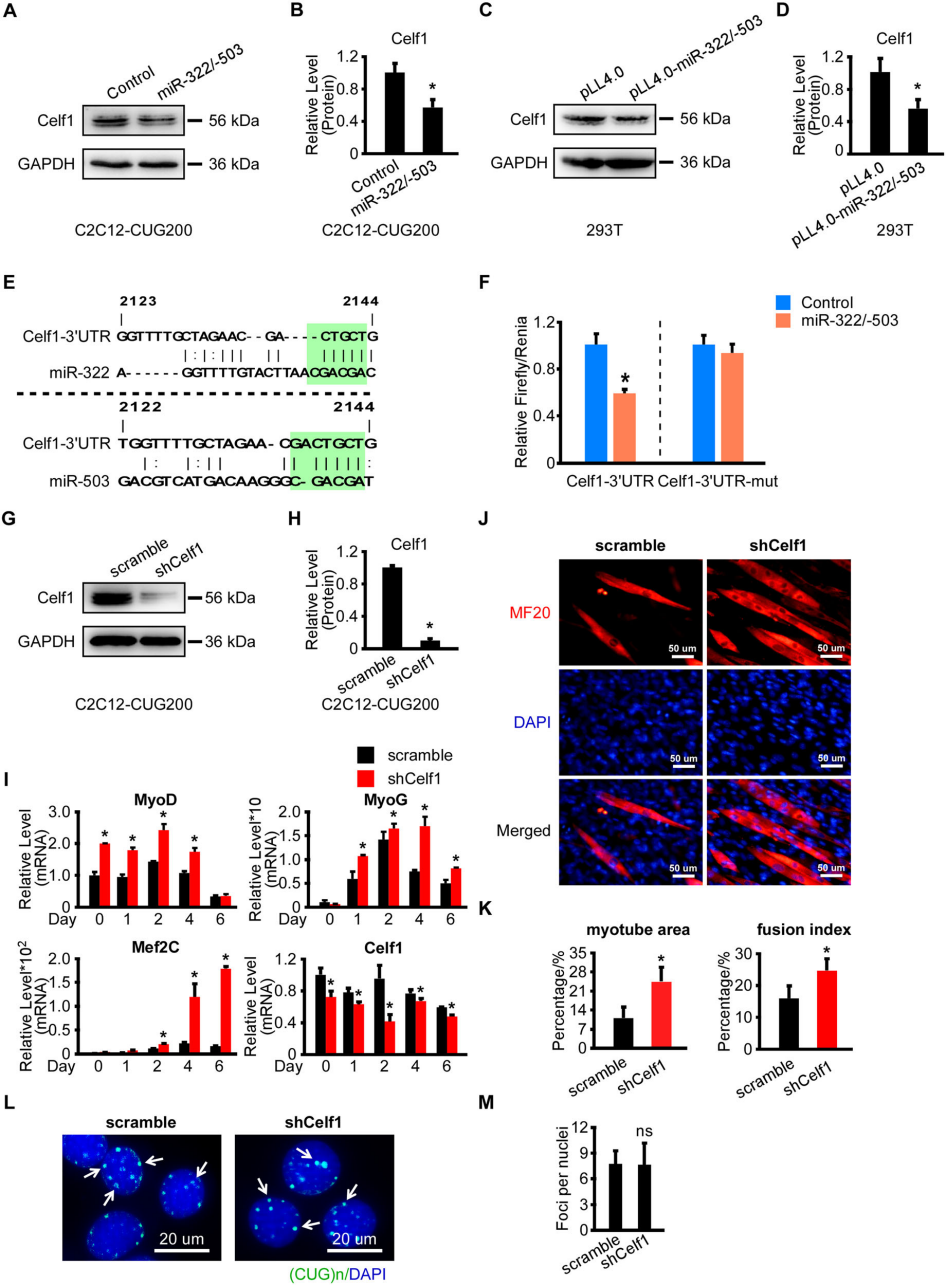
miR-322/-503 targeted Celf1 leading to partial rescue of myoblast differentiation defects but not ribonuclear foci in DM1. **A**, **B** Celf1 protein level was repressed by miR-322/-503 overexpression (OE) in C2C12-CUG200 cells. **C**, **D** Celf1 protein level was repressed by miR-322/-503 OE in HEK293T cells. **E** A mutual binding site of miR-322/-503 on Celf1-3′UTR was predicted by RNA22. **F** miR-322/-503 significantly repressed the relative luciferase activity of luciferase reporter that contained wild-type Celf1-3′UTR. However, the repression was abolished if the predicted binding sites of miR-322/-503 was mutated (Celf1-3′UTR-mut). **G**, **H** The knockdown of Celf1 in C2C12-CUG200 cells was verified by western blot. **I** Celf1 knockdown significantly upregulated the expressions of myogenic markers (MyoD, MyoG, and Mef2C) and downregulated Celf1 expression during C2C12-CUG200 differentiation. All expression levels were normalized to the control group at day 0. **J** Celf1 knockdown promoted myotube formation during C2C12-CUG200 differentiation. Myotube formation was displayed by immunostaining of MF20 on day 6. **K** Celf1 knockdown increased myotube areas (left) and fusion index (right) of C2C12-CUG200 differentiation. **L**, **M** Celf1 knockdown did not repress ribonuclear foci formation in C212-CUG200 cells. The ribonuclear foci were detected with CAG probe RNA FISH. Control, pLL4.0 vector stable transfection; miR-322/-503, pLL4.0-miR-322/-503 stable transfection; pLL4.0, pLL4.0 vector transient transfection; pLL4.0-miR-322/-503, pLL4.0-miR-322/-503 transient transfection; scramble, C2C12-CUG200/scramble cells; shCelf1, C2C12-CUG200/shCelf1 cells; * statistically significant (*p* < 0.05); ns not statistically significant.

We previously reported that miR-322/-503 promotes early cardiac differentiation by targeting Celf1, which was shown to inhibit cardiac differentiation and promote neural differentiation^39^. Inhibition of Celf1 could partially rescue myoblast differentiation defects in DM1 through regulating cell cycle^17^. Therefore, we asked if Celf1 was the downstream target of miR-322/-503 in regulating DM1. In Fig. 3B, Celf1 was downregulated by miR-322/-503 overexpression in the differentiation of C2C12-CUG200 cells. By western blot, we identified marked Celf1 decreases with miR-322/-503 overexpression in both C2C12-CUG200 cells and C2C12 cells (Figs. 4A, B and S1A, S1B). Celf1 was upregulated by miR-322 and miR-503 inhibitors treatment in C2C12 cells (Fig. S1C). The transient transfection of pLL4.0-miR-322/-503 caused a significant reduction of Celf1 in HEK293T cells compared to pLL4.0 control (Fig. 4C, D). With the RNA22 program, we predicted a mutual target site of miR-322/-503 at the 3′UTR of Celf1 (Fig. 4E). According to the prediction, we constructed a wild type and a predicted miR-322/-503 binding site mutated Celf1-3′UTR luciferase assay vector (Celf1-3′UTR and Celf1-3′UTR-mut). By dual-luciferase assay, miR-322/-503 significantly repressed the luciferase activity of Celf1-3′UTR but not Celf1-3′UTR-mut (Fig. 4F). These data proved that miR-322/-503 directly targeted Celf1.

Next, we examined if Celf1 downregulation was able to reproduce miR-322/-503 overexpression in rescuing myoblast defects in DM1. We constructed scramble control and Celf1 knockdown DM1 cell models (namely C2C12-CUG200/scramble and C2C12-CUG200/shCelf1 cells), which were subjected to differentiation. Celf1 knockdown was verified by western blot (Fig. 4G, H). Celf1 knockdown remarkably improved the expressions of myogenic markers (MyoD, MyoG, Mef2C) in DM1, though the improvement levels were relatively lower than miR-322/-503 overexpression (Figs. 3B and 4I). Celf1 knockdown also displayed a modest increase of myotube formation detected by the immunostaining of MF20 (Fig. 4J). The myotube area and fusion index with Celf1 knockdown were significantly improved (24.5% ± 5.2% and 24.7% ± 3.8%) compared to scramble control (11.0% ± 4.2% and 16.0% ± 3.9%), though the improvements were less compared to miR-322/-503 overexpression (Fig. 4K). Moreover, the ribonuclear foci were not altered by Celf1 knockdown, differing from miR-322/-503 overexpression (Figs. 4L, M and S2C). Taken together, Celf1 knockdown partially reproduced the effect of miR-322/-503 in rescuing myoblast differentiation defects in DM1 but did not repress ribonuclear foci formation, suggesting that Celf1 was not the only target of miR-322/-503 in DM1.

### miR-322/-503 directly targeted the expanded CUG repeats

**Fig. 5:**
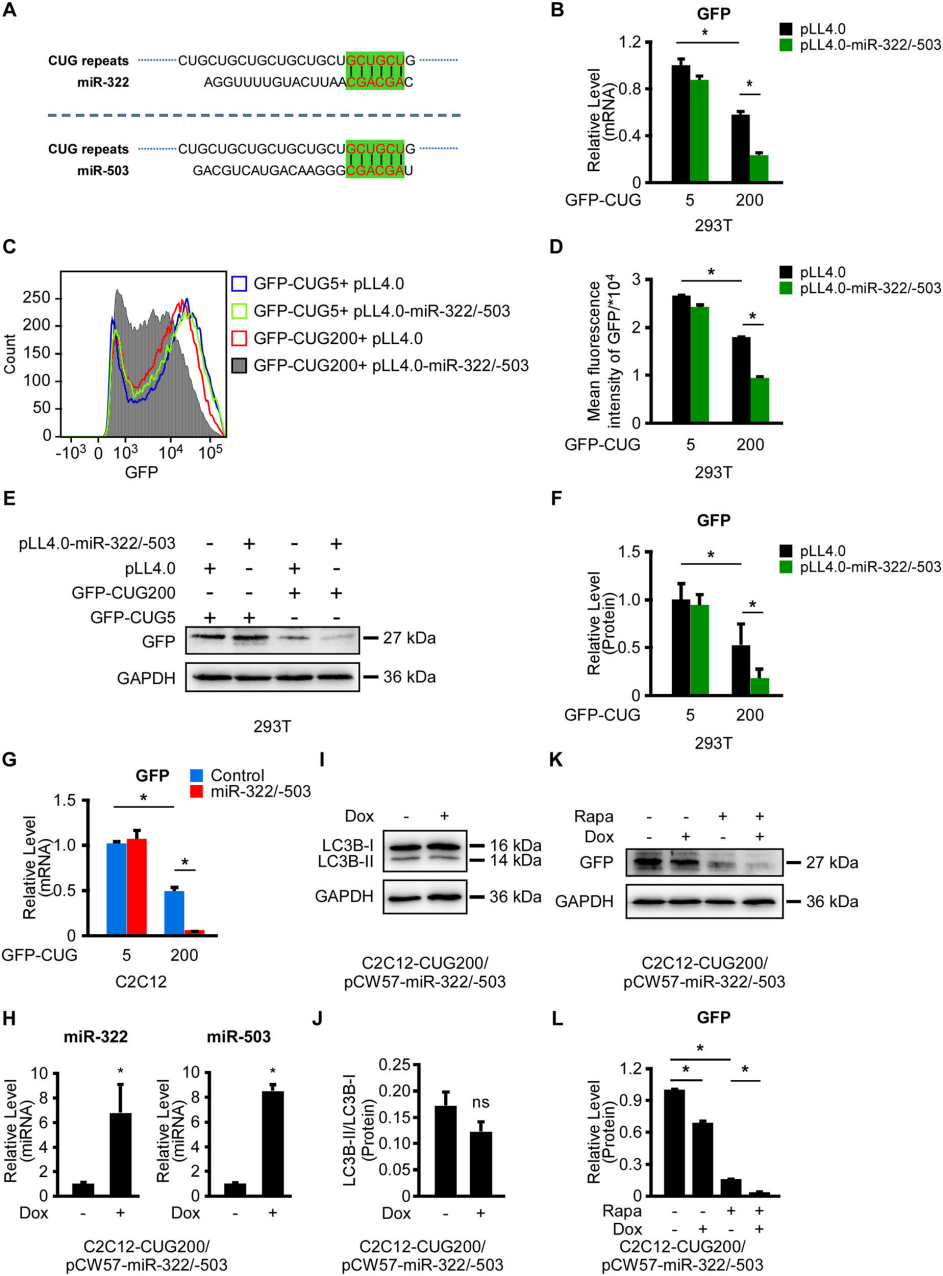
miR-322/-503 specifically antagonized the expanded CUG repeats. GFP-CUG5 and GFP-CUG200 were used as reporters. **A** Schematic diagram showing the predicted bindings of the seed regions of miR-322 and miR-503 to CUG repeats. **B** GFP mRNA level was significantly repressed by miR-322/-503 in the GFP-CUG200 group in HEK293T cells. mRNA level was quantified by RT-qPCR. All expression levels were normalized to the Blank vector/GFP-CUG5 group. **C**, **D** GFP levels were significantly repressed by miR-322/-503 in the GFP-CUG200 group in HKE293T cells. The GFP levels were measured by flow cytometry. Mean GFP fluorescence intensities of four were quantified by the FlowJo v10 software. **E**, **F** GFP protein level was significantly repressed by miR-322/-503 in the GFP-CUG200 group in HKE293T cells. However, the GFP protein level was not affected by miR-322/-503 in the GFP-CUG5 group in HKE293T cells. Protein levels were measured by western blots. **G** GFP mRNA level was significantly repressed by miR-322/-503 in the GFP-CUG200 group in C2C12 cells. However, the GFP mRNA level was not affected by miR-322/-503 in the GFP-CUG5 group in C2C12 cells. mRNA level was quantified by RT-qPCR. All expression levels were normalized to the Blank vector/C2C12-CUG5 group. **H** miR-322/-503 induction by Dox treatment was verified by RT-qPCR in C2C12-CUG200/pCW57-miR-322/-503 cells. All expression levels were normalized to no Dox group. **I**, **J** Autophagy, marked by LC3B-II/LC3B-I ratio, was not affected with Dox-induced miR-322/-503 overexpression in C2C12-CUG200/pCW57-miR-322/-503 cells. LC3B was determined by western blots. **K**, **L** miR-322/-503 regulated GFP levels independent of autophagy in C2C12-CUG200/pCW57-miR-322/-503 cells. All expression levels were normalized to the no treatment group. Control, pLL4.0 vector stable transfection; miR-322/-503, pLL4.0-miR-322/-503 stable transfection; pLL4.0, pLL4.0 vector transient transfection; pLL4.0-miR-322/-503, pLL4.0-miR-322/-503 transient transfection; Rapa rapamycin, Dox Doxycycline. * statistically significant (*p* < 0.05); ns not statistically significant.

As a universal function mechanism, miRNAs induced mRNA degradation and/or translation inhibition via specific binding to their target mRNAs, which relied mainly on the recognition of miRNAs’ seed regions to mRNAs. Surprisingly, we found that the seed regions of both miR-322 and miR-503 were “AGCAGC”, which were complementary to “GCUGCU”, the consisting element of the CUG repeats (Fig. 5A). Thus, we asked if miR-322/-503 directly targeted CUG repeats. Here, we employed the minigene plasmids, GFP-CUG5 and GFP-CUG200, which were used to construct normal and DM1 cell models, as GFP reporters (Fig. 2A). We transiently transduced HEK293T cells with the following plasmid combinations: (i) GFP-CUG5&pLL4.0; (ii) GFP-CUG5&pLL4.0-miR-322/-503; (iii) GFP-CUG200&pLL4.0; and (iv) GFP-CUG200&pLL4.0-miR-322/-503. Basal GFP mRNA and protein levels were downregulated in GFP-CUG200 groups compared to GFP-CUG5 groups, in agreement with a reference^49^ (Fig. 5B, E, F). In GFP-CUG200 groups rather than GFP-CUG5 groups, miR-322/-503 dramatically repressed the GFP mRNA level (Fig. 5B). Next, we evaluated the GFP distributions of those transfection combinations by flow cytometry. Similarly, miR-322/-503 overexpression significantly repressed the GFP distribution in GFP-CUG200 groups but not in GFP-CUG5 groups, which displayed a left shift of GFP peak (Fig. 5C, D). GFP protein levels were also significantly repressed by miR-322/-503 in GFP-CUG200 groups rather than GFP-CUG5 groups. Those results indicated that miR-322/-503 specifically repressed the 200-copy CUG repeats rather than the 5-copy one. We then performed RT-qPCR of GFP on the following stable cell lines: C2C12-CUG5/control, C2C12-CUG5/miR-322/-503, C2C12-CUG200/control, and C2C12-CUG200/miR-322/-503. We identified dramatical repression of GFP mRNA level by miR-322/-503 overexpression in C2C12-CUG200 cells but not in C2C12-CUG5 cells, similar to the results in HEK293T cells (Fig. 5B, G).

A published study uncovered that miR-503 triggered autophagy in esophageal squamous cell carcinoma via the PKA/mTOR pathway^50^. As is known to all, autophagy would affect GFP levels. Thus, we were determined to study if miR-322/-503 affected autophagy in our systems. With different doses of rapamycin treatments, the ratio of LC3B-II/LC3B-I was significantly upregulated in C2C12 cells, suggesting that rapamycin was able to induce autophagy in C2C12 cells (Fig. S3A, B). Next, we constructed Doxycycline (Dox) inducible miR-322/-503 overexpressing C2C12-CUG5 and C2C12-CUG200 cells (namely C2C12-CUG5/pCW57-miR-322/-503 and C2C12-CUG200/pCW57-miR-322/-503 cells). miR-322/-503 overexpression with Dox treatment was verified in both cells by RT-qPCR (Figs. 5H and S3C). LC3B-II/LC3B-I ratios in both cell lines with Dox treatment were not significantly changed, indicating that miR-322/-503 was unable to trigger autophagy in both C2C12-CUG5 and C2C12-CUG200 cells (Figs. 5I, J and S3D, E). Afterward, we performed western blots against GFP in C2C12-CUG5/pCW57-miR-322/-503 and C2C12-CUG200/pCW57-miR-322/-503 cells with the following treatments: (i) no treatment; (ii) Dox only; (iii) rapamycin only; (iv) Dox&rapamycin. Agreeing with our previous results, GFP levels were downregulated in C2C12-CUG200/pCW57-miR-322/-503 cells but not in C2C12-CUG5/pCW57-miR-322/-503 cells (Figs. 5E, F, K, L and S3F, G) with Dox only treatment. With rapamycin only treatment, GFP levels were downregulated in both cell lines compared to no treatment groups. With Dox&rapamycin treatment, GFP levels were even lowered only in C2C12-CUG200/pCW57-miR-322/-503 cells but not in C2C12-CUG5/pCW57-miR-322/-503 cells when compared to rapamycin only treatment groups (Figs. 5K, L and S3F, G). Therefore, the inhibitory effect of miR-322/-503 on GFP in CUG200 groups was autophagy-independent. Together, these results indicated that miR-322/-503 specifically repressed the expanded CUG repeats in DM1, while it hardly reacted with the shorter CUG repeats in normal cells.

**Fig. 6:**
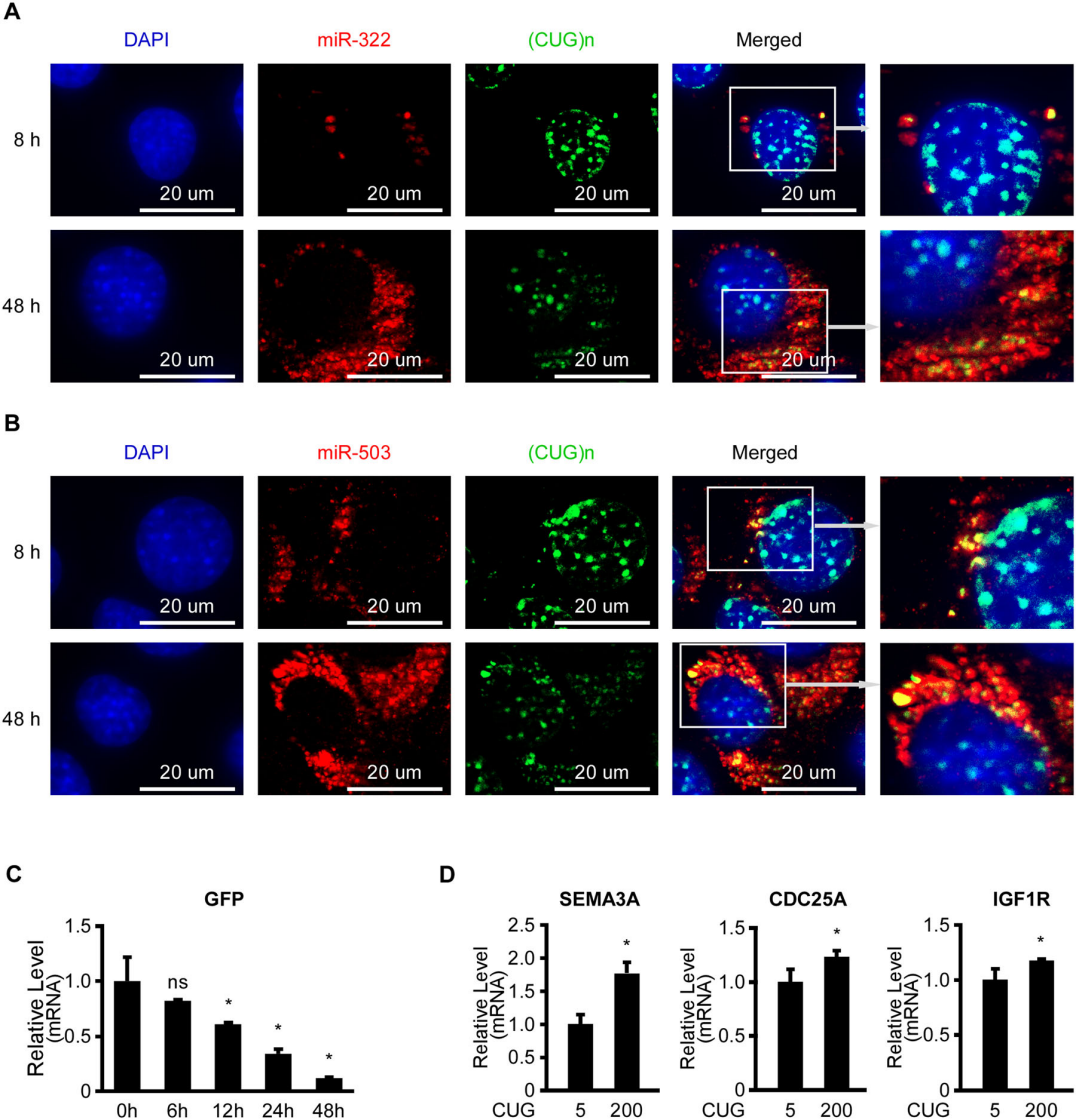
miR-322/-503 directly bound and gradually degraded the expanded CUG repeats. miR-322 (**A**) and miR-503 (**B**) displayed colocalization with the expanded CUG repeats via RNA FISH. The cells at 8 and 48 after Dox induction were used for the RNA FISH staining. **C** GFP mRNA level was measured at 0, 6, 12, 24, 48 h after transient transfection of GFP-CUG200 and miR-322/-503 overexpression plasmids in HEK293T cells through RT-qPCR. All expression levels were normalized to the 0-h group. **D** The levels of SEMA3A, CDC25A, and IGF1R were determined by RT-qPCR in both C2C12-CUG200/pLL4.0-miR-322/-503 and C2C12-CUG5/pLL4.0-miR-322/-503 cells. All expression levels were normalized to the C2C12-CUG5/pLL4.0-miR-322/-503 group. * statistically significant (*p* < 0.05); ns not statistically significant.

To examine if miR-322/-503 was directly bound to the expanded CUG repeats, we performed co-staining of the expanded CUG repeats with either miR-322 or miR-503 via RNA FISH in Dox treated C2C12-CUG200/pCW57-miR-322/-503 cells. There were colocalization of the expanded CUG repeats with either miR-322 or miR-503 in the cytosol near the nucleus at both 8 and 48 h after Dox treatment, which was similar to the previously reported miR-16’s pattern^30^ (Fig. 6A, B). Meanwhile, to exclude the possibility that the dual-overexpression of the expanded CUG repeats and miR-322/-503 in C2C12-CUG200/pCW57-miR-322/-503 cells would interfere with the FISH staining outcomes, we also performed the same FISH staining on the miR-322/-503 overexpressing alone cells (C2C12/pCW57-miR-322/-503 cells) and the CUG200 repeats overexpressing alone cells (C2C12-CUG200/pCW57-miR-322/-503 cells without Dox treatment). The expression patterns of miR-322/-503 and the expanded CUG repeats in those two groups were identical to the dual-overexpressing group (Figs. 6A, B and S4A–D). Moreover, as to the Dox treated C2C12-CUG200/pCW57-miR-322/-503 cells, the colocalization levels of the expanded CUG repeats and miR-322/-503 were higher at 48 h compared to 8 h, while the ribonuclear foci numbers were less with miR-322/-503 induction (Figs. 6A, B and S4E, F), implying that the colocalization resulted in gradual CUG repeats degradation. To prove that, we transiently transfected HEK293T cells with GFP-CUG200&pLL4.0-miR-322/-503 plasmids and harvested samples at 0, 6, 12, 24, 48 h post-transfection. From RT-qPCR, GFP mRNA level was gradually decreased, indicating miR-322/-503 gradually degraded the CUG repeats (Fig. 6C). As miR-322/-503 directly interacts with the expanded CUG repeats, the CUG repeats might act as sponges to absorb miR-322/-503. We performed RT-qPCR of SEMA3A, CDC25A, and IGF1R, which were predicted or proven to be miR-322/-503′s targets, and found those genes were significantly upregulated in C2C12-CUG200/pLL4.0-miR-322/-503 cells compared to C2C12-CUG5/pLL4.0-miR-322/-503 cells, proving that the expanded CUG repeats could absorb miR-322/-503 and indirectly upregulated miR-322/-503 target genes (Fig. 6D). Those results indicated that miR-322/-503 could be directly bound to and gradually degrade the expanded CUG repeats.

### miR-322/-503 corrected aberrant alternative splicing in DM1

**Fig. 7:**
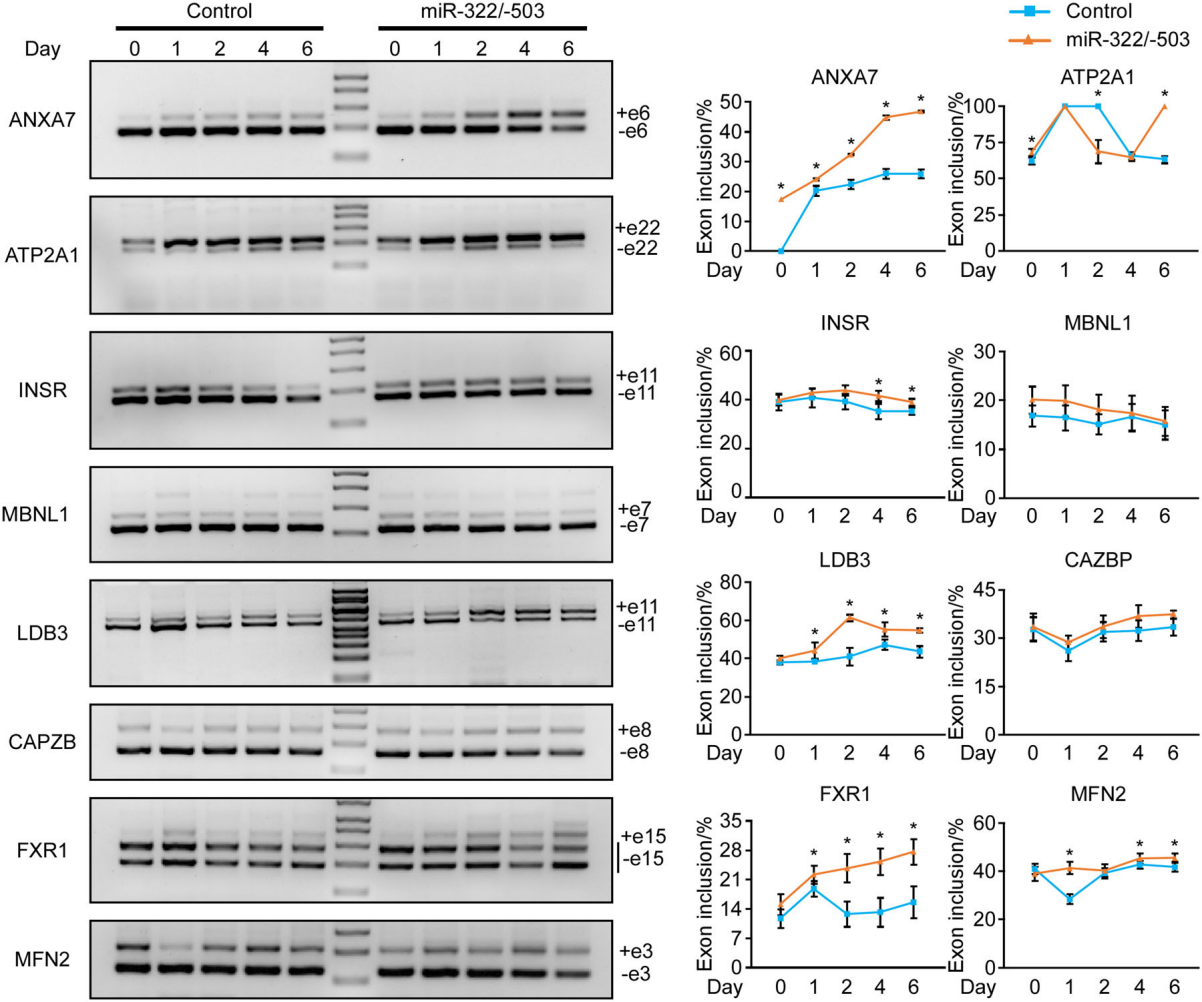
miR-322/-503 corrected aberrant alternative splicing in DM1. **A** The alternative splicing patterns of Anxa7, Atp2a1, Insr, MBNL1, Ldb3, CAPZB, FXR1, and MFN2 were investigated through the course of control and miR-322/-503 overexpression DM1 myoblast differentiation by RT-PCR. The exons that caused band size variations in each gel image were specified. GAPDH served as an internal control. **B** The percentage of exon inclusions of Anxa7, Atp2a1, Insr, MBNL1, Ldb3, CAPZB, FXR1, and MFN2 were plotted according to band densities in (**A**). The optical densities of agarose gel bands were quantified using ImageJ2X software. The optical densities of both exon inclusion and exclusion bands were normalized to corresponding GAPDH bands. The exon inclusion percentage was calculated as follows: exon inclusion% = normalized optical density of exon inclusion/ (normalized optical density of exon inclusion + normalized optical density of exon exclusion). Control, C2C12-CUG200/control cells; miR-322/-503, C2C12-CUG200/miR-322/-503 cells; * statistically significant (*p* < 0.05).

As miR-322/-503 directly targeted and repressed the expanded CUG repeats, we asked if the dysregulated alternative splicing was reversed with ectopic miR-322/-503 expression. We performed RT-PCR to detect the alternative splicing patterns of Anxa7, Atp2a1, Insr, MBNL1, Ldb3, CAPZB, FXR1, and MFN2. Among those genes, the alternative splicing of CAPZB, FXR1, and MFN2 were regulated by Celf1 only. The splicing patterns of these genes were obviously altered in the DM1 model (CUG200) vs. normal (CUG5). The inclusion of Anxa7 exon6, Insr exon11, MBNL1 exon7, Ldb3 exon11, CAPZB exon8, FXR1 exon15, and MFN2 exon 3 was obviously downregulated, while the inclusion of Atp2a1 exon22 was slightly upregulated through myoblast differentiation of the DM1 C2C12 cells (Fig. S5). With the overexpression of miR-322/-503, the alternative splicing patterns of these genes were reversed (Fig. 7). Together, these results suggested that miR-322/-503 corrected dysregulated alternative splicing.

**Fig. 8:**
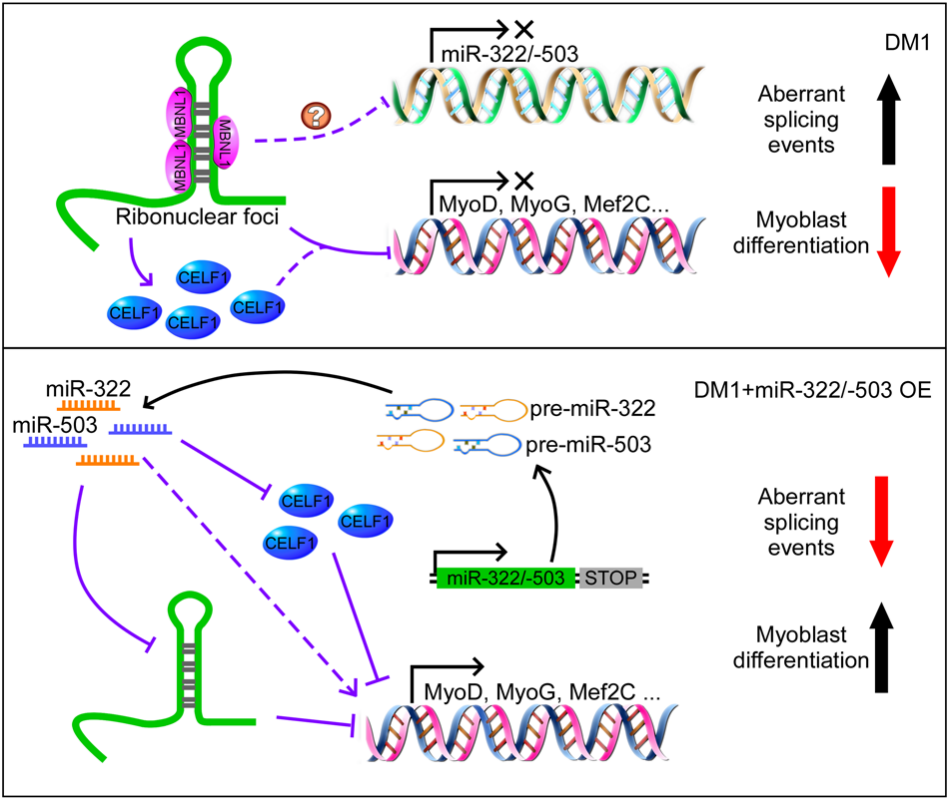
A working model of miR-322/-503 in DM1. miR-322/-503, required by normal myoblast differentiation, was downregulated in DM1 myoblast differentiation and patient serums. Ectopic miR-322/-503 expression rescued myoblast differentiation defects, ribonuclear foci, and aberrant alternative splicing in DM1 myoblast mainly through targeting the expanded CUG repeats. Meanwhile, Celf1 as an alternative miR-322/-503 target only accounts for the partial rescue of myoblast differentiation defects. This figure was drawn with Pathway Builder 2.0.

In conclusion, we here discovered that miR-322/-503 could rescue myoblast defects in DM1 by directly targeting the expanded CUG repeats. By gain-and loss-of-function analysis, we found that miR-322/-503 was required by normal myoblast differentiation. In the DM1 cell model, ectopic miR-322/-503 expression rescued myoblast differentiation defects, ribonuclear foci, and aberrant alternative splicing. Celf1 and the expanded CUG repeats were proven to be miR-322/-503’s targets. Moreover, miR-322/-503 specifically targeted the expanded CUG repeats in DM1 rather than in normal cells, resulting in ribonuclear foci dissolution. The specificity implied a potential therapeutic function of miR-322/-503 against DM1, which needed further examination (Fig. 8).

## Discussion

DM1 is one of the most prevalent adult inherited diseases. The fundamental cause of DM1 is the expanded CUG repeats, which results in spliceopathy and muscular dystrophy. Many miRNAs have been reported to function in DM1. In this study, we established the required role of miR-322/-503 in normal myoblast differentiation and its therapeutic potential of rescuing muscular defects in DM1 through directly targeting the expanded CUG repeats.

Firstly, we discovered that miR-322/-503 is required by the normal myoblast differentiation process. In the loss-of-function test, although the losses of miR-322 and miR-503 both repressed myoblast differentiation, the loss of miR-503 caused more severe differentiation repression, suggesting miR-503 might be relatively more important in regulating myoblast differentiation. In the gain-of-function test, we identified a significant improvement of myoblast differentiation with miR-322/-503 overexpression. In previous studies, miR-322/-503′s role in myoblast differentiation was in arguing. One study stated that miR-322/-503 promoted myoblast differentiation by targeting Cdc25A^40^, which agreed with our findings. However, the other recent report argued that miR-322 repressed myoblast differentiation by targeting SETD3^41^. In this paper, the authors used a myoblast DM without insulin supplement, differing from our culture conditions. According to references, miR-322/-503 was reported to interact with insulin involved pathways^38,51^, and directly regulate insulin resistance^52^. Therefore, the difference in DM might bias the role of miR-322/-503 in myoblast differentiation.

To study myoblast differentiation in DM1, we adopted C2C12-CUG5 cells as the normal model and C2C12-CUG200 cells as the DM1 model. C2C12-CUG200 cells displayed defective myoblast differentiation, ribonuclear foci, and aberrant alternative splicing, which reproduced the pathologic phenotypes of DM1 skeletal muscle. In C2C12-CUG200 cell differentiation, we noticed a dramatic decrease of miR-322/-503. Similarly, we found that the levels of miR-424 (miR-322 orthologue) and miR-503 level also tended to be lower (although not significantly) in DM1 patient serums through analyzing a publicly available RT-qPCR data of healthy individuals (*n* = 26) and DM1 patients (*n* = 24) from a published reference (data not shown)^53^, suggesting that miR-424/-503 might be downregulated also in DM1 myoblast differentiation in humans. The mechanism of how miR-322/-503 was downregulated in DM1 was still unknown. The possible reason was the disturbed miRNA biogenesis in DM1. It was reported that miR-1 was downregulated in the heart because of the impaired Dicer activity resulting from MBNL1 sequestration in DM1, suggesting that the Dicer involved miRNA processing might be dysregulated in DM1^29^. That might be the cause of miR-322/-503 dysregulation in DM1 as well. This hypothesis needed experiments to verify, which was beyond the scope of this study.

Next, we found that ectopic miR-322/-503 expression successfully rescued the myoblast differentiation defects, ribonuclear foci formation, and aberrant alternative splicing in the DM1 cell model through targeting the expanded CUG repeats and Celf1. Celf1 is an important pathogenic factor in DM1. Transgenic Celf1 overexpression in mice heart and skeletal muscle was able to display similar DM1 phenotypes^13,14^. Since our previous study suggested that miR-322/-503 targeted Celf1 in regulating cardiac differentiation^39^, we asked if miR-322/-503 rescued muscular defects through targeting Celf1. Celf1 was proven to be miR-322/-503’s target and its knockdown improved myoblast differentiation defects in DM1 to some extent, agreeing with the previous reference^17,39^. However, the Celf1 knockdown was unable to repress the formation of ribonuclear foci, differing from the outcomes of miR-322/-503 overexpression in DM1, suggesting that other miR-322/-503 involved regulatory mechanisms existed in DM1. Then we revealed that miR-322/-503 directly targeted the expanded CUG repeats. Through expression, reporter, and colocalization assays, we found that miR-322/-503′s targeting the CUG repeats only restricted to the expanded ones in DM1 rather than in normal cells.

Aberrant alternative splicing was caused by the expanded CUG repeats in DM1, which directly mediated the disease phenotypes of DM1. As we proved that miR-322/-503 targeted the expanded CUG repeats, we wondered if the miR-322/-503 corrected aberrant alternative splicing in DM1. Through RT-PCR, we found the aberrant splicing patterns of Anxa7, Atp2a1, Insr, MBNL1, Ldb3, CAPZB, FXR1, and MFN2 in DM1 were rescued with the ectopic miR-322/-503 expression. Among those genes, the alternative splicing of CAPZB, FXR1, and MFN2 were specifically regulated by Celf1. These suggested that miR-322/-503 rescued aberrant alternative splicing in DM1 via targeting both the expanded CUG repeats and Celf1.

In summary, we here discovered that miR-322/-503, which is required by normal myoblast differentiation, could rescue muscular defects in DM1 mainly by targeting the expanded CUG repeats. miR-322/-503’s targeting toward CUG repeats displayed high specificity to the length in DM1 rather than normal cases. With those results, future studies on whether miR-322/-503 could be applied to DM1 therapy would be attractive.

## Supplementary information

Table S1. The sequences of RT-qPCR primers

Table S2. The sequences of primers used to detect alternative splicing

Supplementary Figure Legends

Figure S1. Celf1 was negatively regulated by miR-322/-503 in C2C12 cells.

Figure S2. MBNL1 immunostaining results.

Figure S3. miR-322/-503 had no effect on autophagy in C2C12-CUG5 cells.

Figure S4. RNA foci formation was decreased and miRNA-CUG repeats colocalization was increased with miR-322/-503 induction for 8 and 48 hours in C2C12-CUG200/pCW57-miR-322/-503 cells.

Figure S5. DM1 caused aberrant alternative splicing.

## Data Availability

The datasets used in the current study are available on reasonable request.
