# Supplementary material for: miR-322/-503 rescues myoblast defects in myotonic dystrophy type 1 cell model by targeting CUG repeats
Source: Cell Death Dis. 2020 Oct 22;11(10):891. doi: 10.1038/s41419-020-03112-6 (PMC7582138; doi:10.1038/s41419-020-03112-6)
Supplement: Supplementary file 1 — Table S1. The sequences of RT-qPCR primers [file 41419_2020_3112_MOESM1_ESM.docx]

| Primers | Sequences (5’ to 3’) |
| --- | --- |
| mGAPDH-F | CAAGCTCATTTCCTGGTATGACAA |
| mGAPDH-R | GGGATAGGGCCTCTCTTGCT |
| MyoD-F | AGTGAATGAGGCCTTCGAGA |
| MyoD-R | GCATCTGAGTCGCCACTGTA |
| MyoG-F | ACTCCCTTACGTCCATCGTG |
| MyoG-R | CAGGACAGCCCCACTTAAAA |
| Mef2C-F | CGGTGTCGTCAGTTGTATGG |
| Mef2C-R | TGCAGTAGATATGCGGCTTG |
| Celf1-F | CAGTTCAGGGTCTTCACCGA |
| Celf1-R | GGCTTCCATGGTACTCCCAG |
| miR-322-F | AGCAGCAGCAATTCATGT |
| miR-322-R | GGTCCAGTTTTTTTTTTTTTTTCCAA |
| miR-503-F | AGCAGCGGGAACAGT |
| miR-503-R | CCAGTTTTTTTTTTTTTTTCTGCAGT |
| U6-F | CTCGCTTCGGCAGCACA |
| U6-R | AACGCTTCACGAATTTGCGT |
| hGAPDH-F | ACCATCTTCCAGGAGCGAGAT |
| hGAPDH-R | ATGACGAACATGGGGGCATC |
| GFP-F | GAACCGCATCGAGCTGAA |
| GFP-R | TGCTTGTCGGCCATGATATAG |
| mIGF1R-F | GTGGGGGCTCGTGTTTCTC |
| mIGF1R-R | GATCACCGTGCAGTTTTCCA |
| mCDC25A-F | ACAGCAGTCTACAGAGAATGGG |
| mCDC25A-R | GATGAGGTGAAAGGTGTCTTGG |
| mSEMA3A-F | ATCAGTGGGTGCCTTACCAA |
| mSEMA3A-R | GCCAAATGTTTTACTGGGACA |

**Table S1. The sequences of RT-qPCR primers**
