# Supplementary material for: miR-322/-503 rescues myoblast defects in myotonic dystrophy type 1 cell model by targeting CUG repeats
Source: Cell Death Dis. 2020 Oct 22;11(10):891. doi: 10.1038/s41419-020-03112-6 (PMC7582138; doi:10.1038/s41419-020-03112-6)
Supplement: Supplementary file 2 — Table S2. The sequences of primers used to detect alternative splicing [file 41419_2020_3112_MOESM2_ESM.docx]

| Primers | Sequences (5’ to 3’) | Exons Detected |
| --- | --- | --- |
| mGAPDH-F | CAAGCTCATTTCCTGGTATGACAA | NA |
| mGAPDH-R | GGGATAGGGCCTCTCTTGCT |  |
| ANXA7-F | TCAGTACCCTGGAGGACAAG | 6 |
| ANXA7-R | CTCTGGTCATTGGAACGGTTAG |  |
| INSR-F | CAGATCCTGAAGGAGCTGGAGG | 11 |
| INSR-R | GGGCACAATGGTAGAGGAGACG |  |
| MBNL1-F | GCTGCCCAATACCAGGTCAAC | 7 |
| MBNL1-R | TGGTGGGAGAAATGCTGTATGC |  |
| ATP2A1-F | GCTCATGGTCCTCAAGATCTCAC | 22 |
| ATP2A1-R | GGGTCAGTGCCTCAGCTTTG |  |
| LDB3-F | GGAAGATGAGGCTGATGAGTGG | 11 |
| LDB3-R | TGCTGACAGTGGTAGTGCTCTTTC |  |
| CAPZB-F | GCACGCTGAATGAGATCTACTTTG | 8 |
| CAPZB-R | CCGGTTAGCGTGAAGCAGAG |  |
| FXR1-F | GATAATACAGAATCCGATCAG | 15 |
| FXR1-R | CTGAAGGACCATGCTCTTCAATCAC |  |
| MFN2-F | TTCTGACTCCAGCCATGTCC | 3 |
| MFN2-R | CTTCAGCCATGTGTCGCTTA |  |

**Table S2. The sequences of primers used to detect alternative splicing**
