# Supplementary material for: miR-322/-503 rescues myoblast defects in myotonic dystrophy type 1 cell model by targeting CUG repeats
Source: Cell Death Dis. 2020 Oct 22;11(10):891. doi: 10.1038/s41419-020-03112-6 (PMC7582138; doi:10.1038/s41419-020-03112-6)
Supplement: Supplementary file 3 — Supplementary Figure Legends [file 41419_2020_3112_MOESM3_ESM.docx]

**Supplementary Figure Legends**

**Figure S1. Celf1 was negatively regulated by miR-322/-503 in C2C12 cells.** (**A, B**) Celf1 protein level was repressed by miR-322/-503 overexpression (OE) in C2C12 cells. (**C**) Celf1 mRNA level was upregulated with the treatment of miR-322/-503 inhibitors (mirKD-322 and mirKD-503) in C2C12 cells. mirKD-Ctrl, C2C12/mirKD-Ctrl cells; mirKD-322, C2C12/mirKD-322 cells; mirKD-503, C2C12/mirKD-503 cells; Control, C2C12/control cells; miR-322/-503, C2C12/miR-322/-503 cell lines; *, statistically significant (p<0.05).

**Figure S2. MBNL1 immunostaining results.** (**A**) MBNL1 immunostaining in C2C12-CUG5 and C2C12-CUG200 cells. (**B**) MBNL1 immunostaining in C2C12-CUG200/pLL4.0 and C2C12-CUG200/pLL4.0-miR-322/-503 cells. (**C**) MBNL1 immunostaining in C2C12-CUG200/scramble and C2C12-CUG200/shCelf1 cells. (**D**) MBNL1 immunostaining in C2C12-CUG200/pLL4.0 and C2C12-CUG200/pLL4.0-miR-322/-503 cells that were captured with lower fluorescence threshold to show diffused MBNL1. (E) The total fluorescence intensities of MBNL1 in C2C12-CUG200/pLL4.0 and C2C12-CUG200/pLL4.0-miR-322/-503 cells were measured by ImageJ2X. CUG5, C2C12-CUG5 cells; CUG200, C2C12-CUG200 cells; Control, C2C12-CUG200/control cells; miR-322/-503, C2C12-CUG200/miR-322/-503 cells; scramble, C2C12-CUG200/scramble cells; shCelf1, C2C12-CUG200/shCelf1 cells.

**Figure S3. miR-322/-503 had no effect on autophagy in C2C12-CUG5 cells.** (**A, B**) rapamycin treatment successfully triggered autophagy, indicated by elevated LC3B-II/LC3B-1 ratios. (**C**) Doxycycline treatment-induced overexpression of miR-322/-503 in C2C12-CUG5/pCW57-miR-322/-503. (**D, E**) miR-322/-503 overexpression did not promote autophagy in C2C12-CUG5/pCW57-miR-322/-503 cells, indicated by unchanged LC3B-II/LC3B-1 ratios. (**F, G**) miR-322/-503 had no effect on GFP levels in C2C12-CUG5/pCW57-miR-322/-503 cells. All expression levels were normalized to the no treatment group. Rapa, rapamycin; Dox, Doxycycline; *, statistically significant (p<0.05); ns, not statistically significant.

**Figure S4. RNA foci formation was decreased and miRNA-CUG repeats colocalization was increased with miR-322/-503 induction for 8 and 48 hours in C2C12-CUG200/pCW57-miR-322/-503 cells.** miR-322 (**A**) and miR-503 (**B**) were induced in C2C12/pCW57-miR-322/-503 cells with Dox treatment. The cells at 8 and 48 hours after Dox induction were used for the RNA FISH staining. miR-322 (**C**) and miR-503 (**D**) were invisible without Dox induction in C2C12-CUG200/pCW57-miR-322/-503 cells, while RNA foci structures were clearly displayed. (**E**) statistical analysis of RNA foci numbers with Dox treatment on C2C12-CUG200/pCW57-miR-322/-503 cells for 8 and 48 hours. (**F**) statistical analysis of co-localization ratio with Dox treatment on C2C12-CUG200/pCW57-miR-322/-503 cells for 8 and 48 hours using ImageJ2X software. Dox, Doxycycline; *, statistically significant (p<0.05); ns, not statistically significant.

**Figure S5. DM1 caused aberrant alternative splicing.** (**A**) The alternative splicing patterns of Anxa7, Atp2a1, Insr, MBNL1, Ldb3, CAPZB, FXR1, and MFN2 were investigated through the course of control and DM1 myoblast differentiation by RT-PCR. The exons that caused band size variations in each gel image were specified. GAPDH served as an internal control. (**B**) The percentage of exon inclusions of Anxa7, Atp2a1, Insr, MBNL1, Ldb3, CAPZB, FXR1, and MFN2 were plotted according to (**A**). The optical densities of agarose gel bands were quantified using ImageJ2X software. The optical densities of both exon inclusion and exclusion bands were normalized to corresponding GAPDH bands. The exon inclusion percentage was calculated as follows: exon inclusion%= normalized optical density of exon inclusion/ (normalized optical density of exon inclusion + normalized optical density of exon exclusion). CUG5, C2C12-CUG5 cells; CUG200, C2C12-CUG200 cells; *, statistically significant (p<0.05).
