# Supplementary figures and images for: miR-322/-503 rescues myoblast defects in myotonic dystrophy type 1 cell model by targeting CUG repeats
Source: Cell Death Dis. 2020 Oct 22;11(10):891. doi: 10.1038/s41419-020-03112-6 (PMC7582138; doi:10.1038/s41419-020-03112-6)

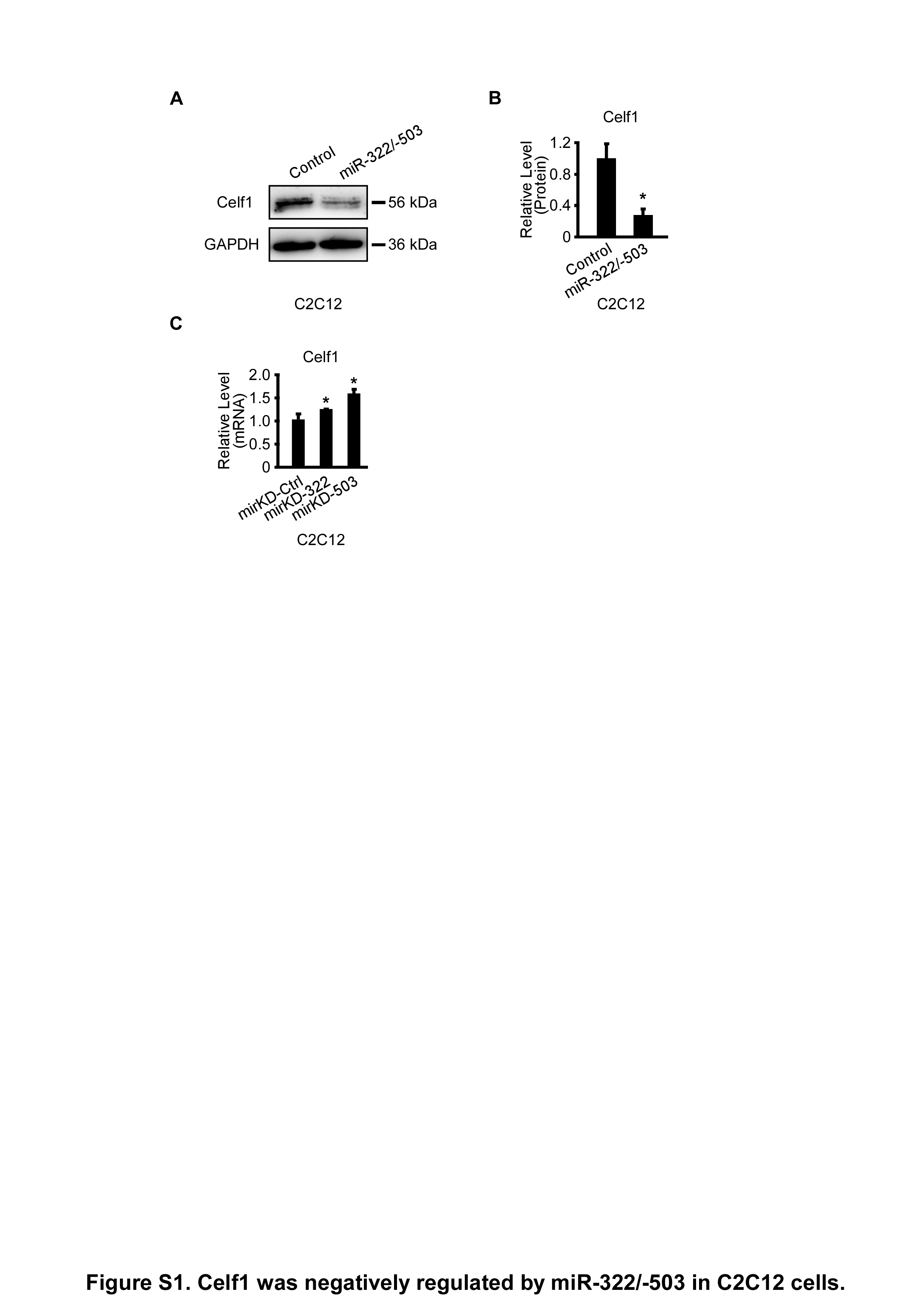

Supplement: Supplementary file 4 — Figure S1. Celf1 was negatively regulated by miR-322/-503 in C2C12 cells. [file 41419_2020_3112_MOESM4_ESM.tif]

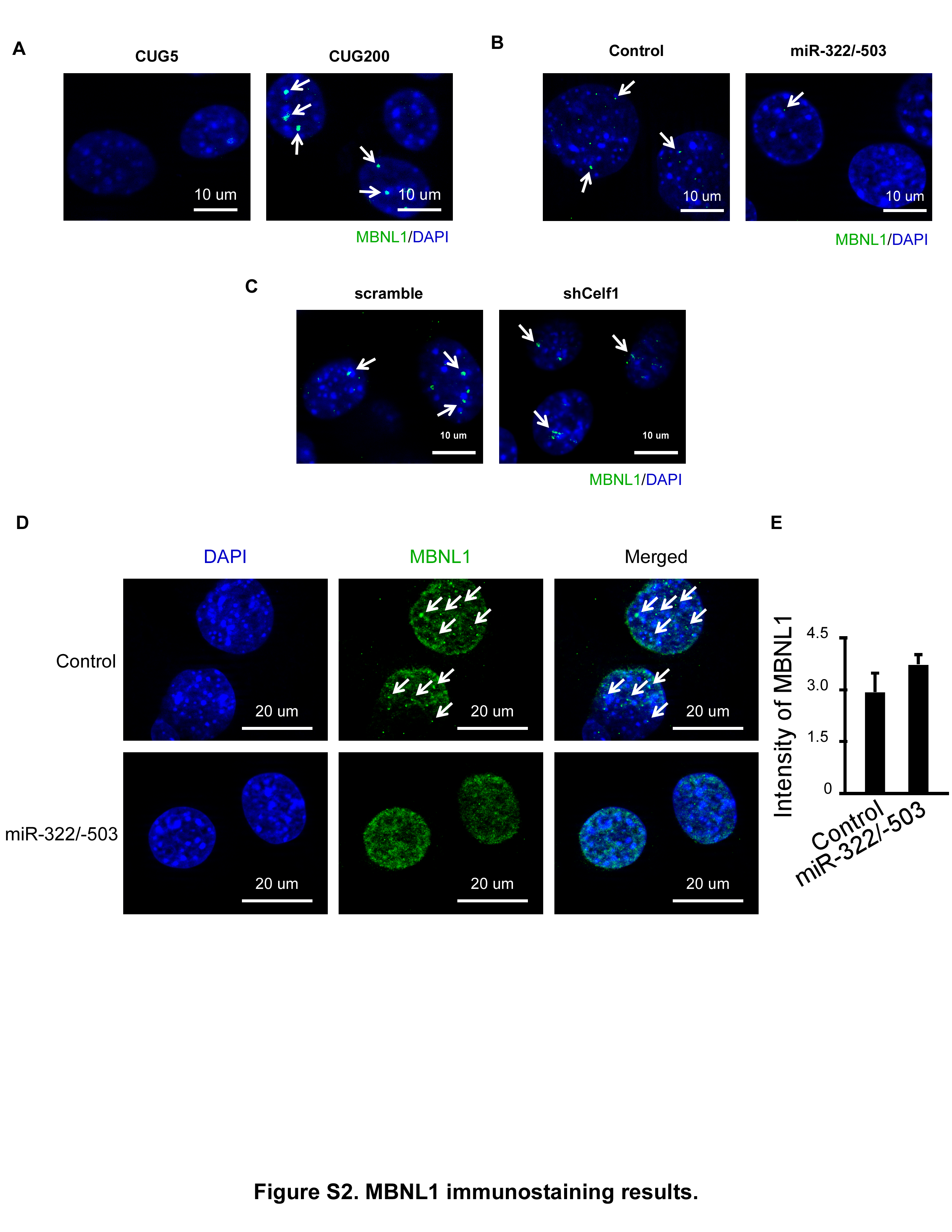

Supplement: Supplementary file 5 — Figure S2. MBNL1 immunostaining results. [file 41419_2020_3112_MOESM5_ESM.tif]

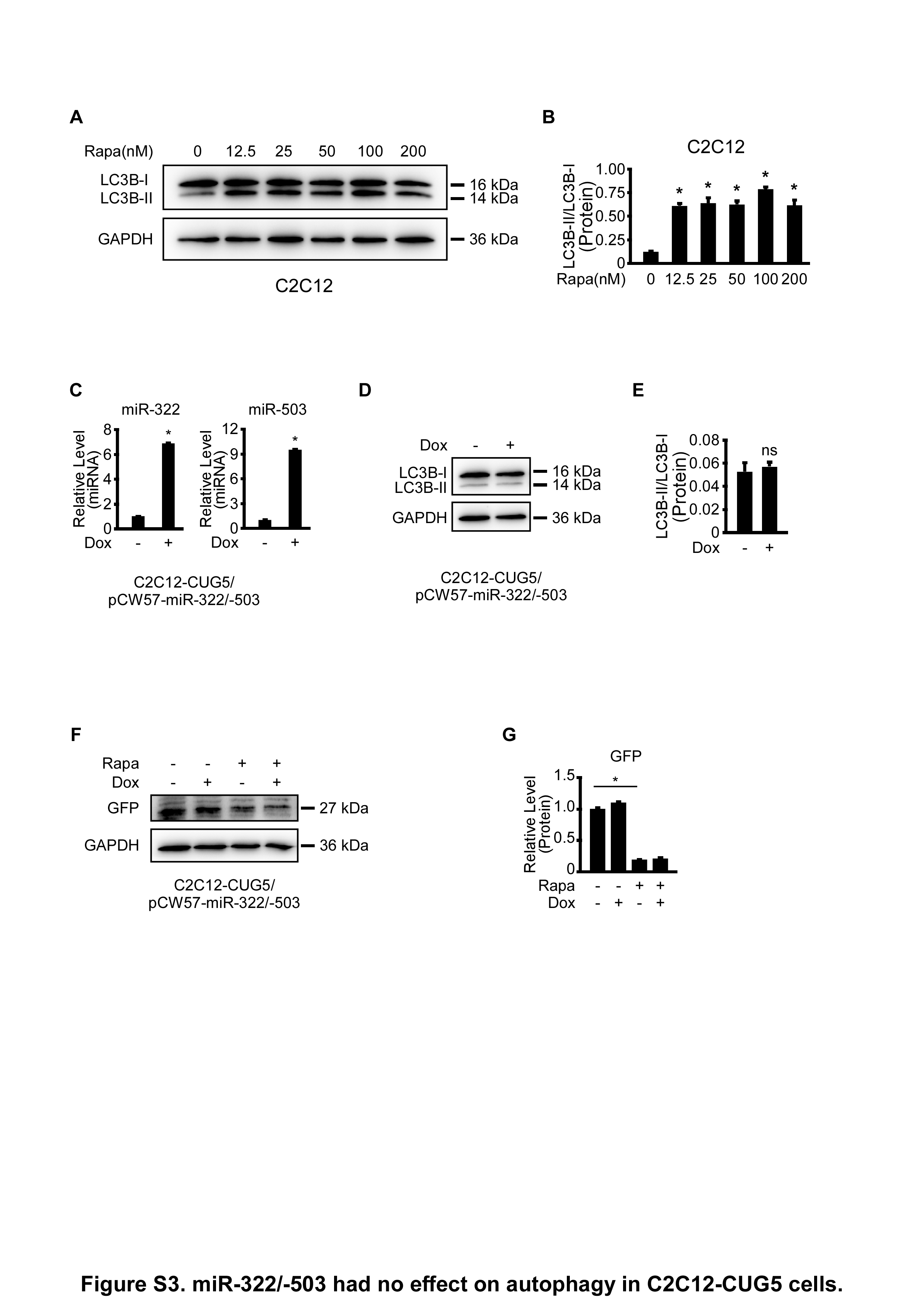

Supplement: Supplementary file 6 — Figure S3. miR-322/-503 had no effect on autophagy in C2C12-CUG5 cells. [file 41419_2020_3112_MOESM6_ESM.tif]

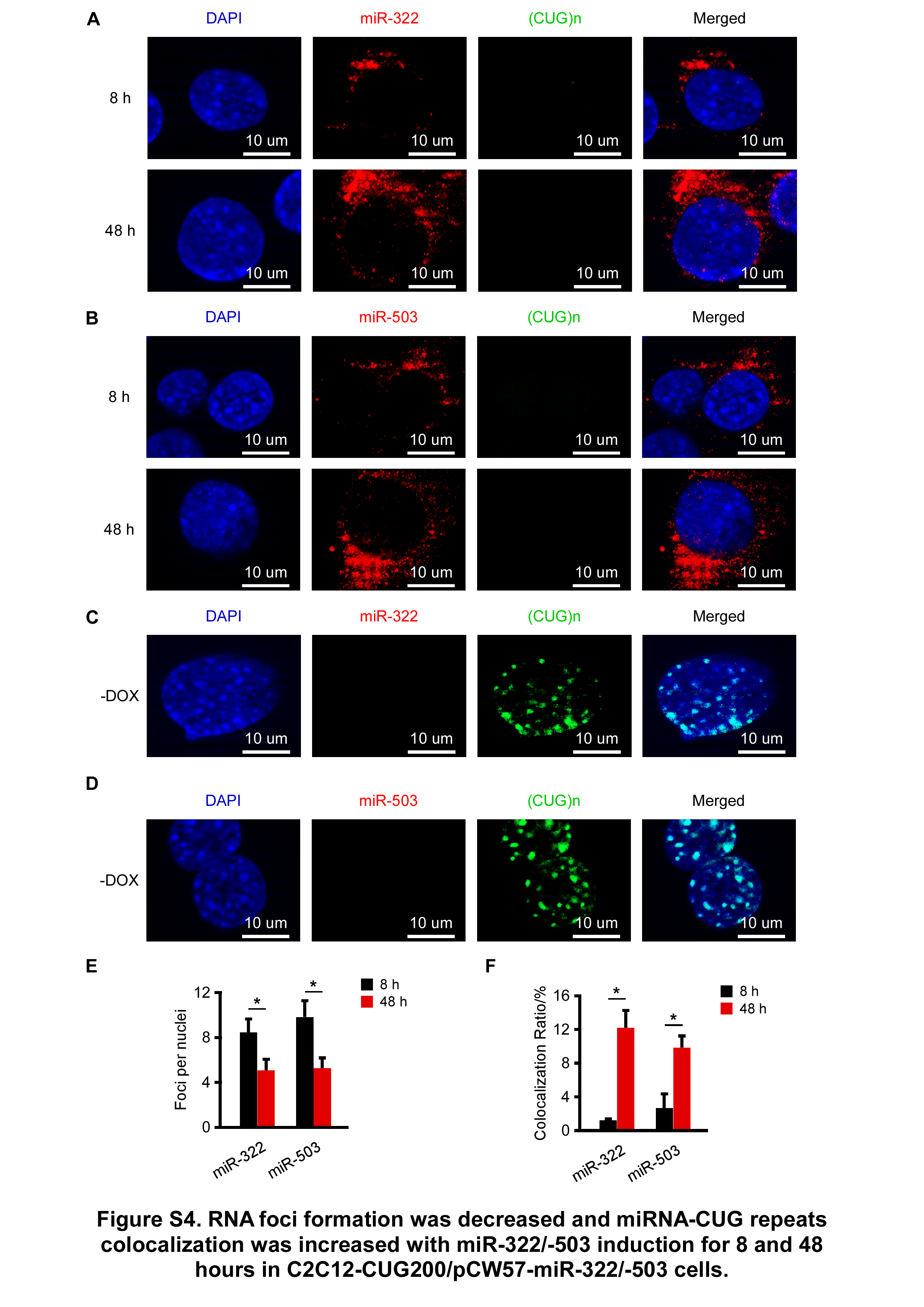

Supplement: Supplementary file 7 — Figure S4. RNA foci formation was decreased and miRNA-CUG repeats colocalization was increased with miR-322/-503 induction for 8 and 48 hours in C2C12-CUG200/pCW57-miR-322/-503 cells. [file 41419_2020_3112_MOESM7_ESM.tif]

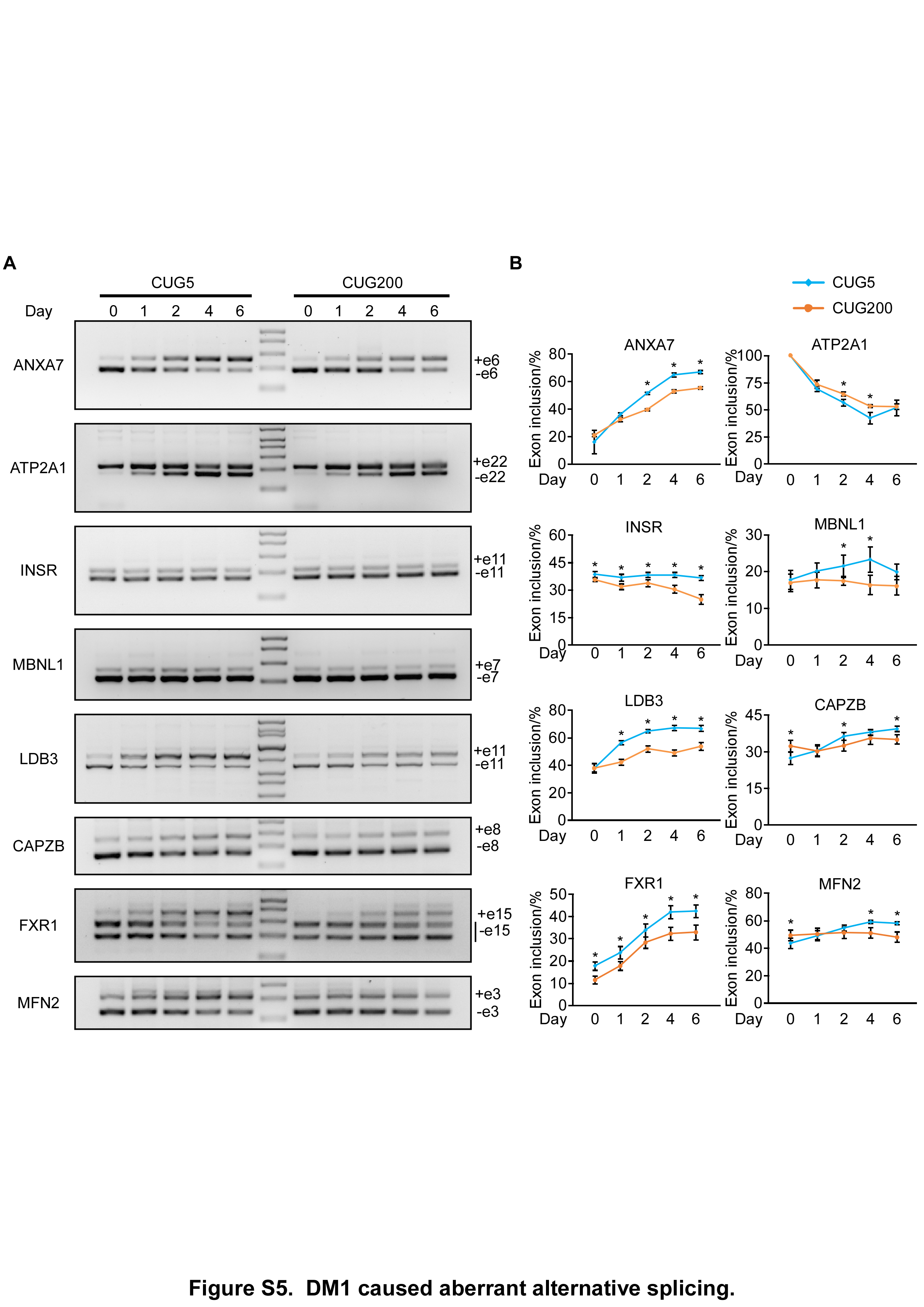

Supplement: Supplementary file 8 — Figure S5. DM1 caused aberrant alternative splicing. [file 41419_2020_3112_MOESM8_ESM.tif]
